# Ultraflexible organic amplifier with biocompatible gel electrodes

**DOI:** 10.1038/ncomms11425

**Published:** 2016-04-29

**Authors:** Tsuyoshi Sekitani, Tomoyuki Yokota, Kazunori Kuribara, Martin Kaltenbrunner, Takanori Fukushima, Yusuke Inoue, Masaki Sekino, Takashi Isoyama, Yusuke Abe, Hiroshi Onodera, Takao Someya

**Affiliations:** 1 Department of Electrical and Electronic Engineering, The University of Tokyo, 7-3-1 Hongo Bunkyo-ku, Tokyo 113-8656, Japan; 2 The Institute of Scientific and Industrial Research, Osaka University, 8-1, Mihogaoka, Ibaraki, Osaka 567-0047, Japan; 3 Department of Applied Physics, The University of Tokyo, 7-3-1 Hongo, Bunkyo-ku, Tokyo 113-8656, Japan; 4 Soft Matter Physics, Linz Institute of Technology LIT, Johannes Kepler University Linz, Altenbergerstrasse 69, Linz 4040, Austria; 5 Chemical Resource Laboratory, Tokyo Institute of Technology, 4259R1-1, Nagatsuda, Midoriku, Yokohama, Kanagawa 226-8503, Japan; 6 Department of Biomedical Engineering, Graduate School of Medicine, The University of Tokyo, 7-3-1 Hongo, Bunkyo-ku, Tokyo 113-8656, Japan; 7 Photon Science Center, The University of Tokyo, 7-3-1 Hongo, Bunkyo-ku, Tokyo 113-8656, Japan

## Abstract

*In vivo* electronic monitoring systems are promising technology to obtain biosignals with high spatiotemporal resolution and sensitivity. Here we demonstrate the fabrication of a biocompatible highly conductive gel composite comprising multi-walled carbon nanotube-dispersed sheet with an aqueous hydrogel. This gel composite exhibits admittance of 100 mS cm^−2^ and maintains high admittance even in a low-frequency range. On implantation into a living hypodermal tissue for 4 weeks, it showed a small foreign-body reaction compared with widely used metal electrodes. Capitalizing on the multi-functional gel composite, we fabricated an ultrathin and mechanically flexible organic active matrix amplifier on a 1.2-μm-thick polyethylene-naphthalate film to amplify (amplification factor: ∼200) weak biosignals. The composite was integrated to the amplifier to realize a direct lead epicardial electrocardiography that is easily spread over an uneven heart tissue.

## Abstract (web summary)

Flexible electronics promise the opportunity to monitor biological activity via implanted devices. Here, the authors develop a biocompatible conductive carbon nanotube/gel composite and couple it with an ultrathin flexible amplifier, enabling *in vivo* measurement of epicardial electrocardiogram signals.

## Graphical abstract

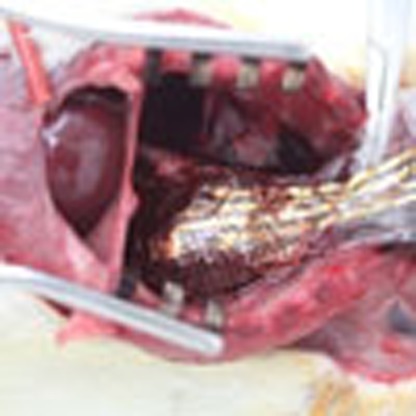

An implantable electronic system that monitors *in vivo* biological signals is expected to play an important role in realizing next-generation medical electronics and in deeply understanding biological systems. By using non-invasive medical instruments such as magnetic resonance imaging, ultrasound and X-ray systems, various types of biological information can be obtained from outside the body. However, by going inside the body (*in vivo*), abundant biological information can be measured with higher spatial and temporal resolution and sensitivity. One of the ultimate goals of *in vivo* monitoring systems is to elucidate biological activities at the organ level, such as in the brain and heart, with high spatial and temporal resolution over a large area.

Long-term stability and reliability are among the biggest challenges for *in vivo* electronic systems. In fact, realizing long-term *in vivo* monitoring is very difficult, because monitoring biosignals requires sensitivity on the order of microvolts to millivolts, which is made further difficult because of the inherently wet and deformable surfaces of biological tissues. Although a pacemaker for heart stimulation and a probe of deep brain stimulation for supressing epilepsy have a lifetime exceeding 10 years in the body, they simply apply high voltages (typically ∼5 V) to stimulate heartbeats and neural networks, respectively, and do not perform detection. To increase *in vivo* monitoring periods with microvolt sensitivity, improving the long-term stability, reliability (especially the signal-to-noise ratio (SNR)) and biocompatibility of electrodes used in electric probes that directly touch the surface of biological tissues is important. Replacing conventional hard metal electrodes with soft conducting biocompatible materials is a promising solution to obtain reliable large-area mechanical and electrical contacts at the bioelectrode interfaces. Furthermore, integration of soft conductive biocompatible materials with ultraflexible electronic amplifier is indispensable in realizing *in vivo* monitoring periods with sensitivity on the order of microvolts to millivolts.

Here we demonstrate the fabrication of a biocompatible, ultraflexible and thin-film organic amplifier using biocompatible highly conductive gel electrodes with organic transistor-based circuits. The gel electrodes comprise multi-walled carbon nanotube (CNT)-dispersed sheet with an aqueous hydrogel. This gel electrode exhibits admittance of 100 mS cm^−2^ and maintains high admittance even in a low-frequency range. On implantation into a living hypodermal tissue for 4 weeks, it showed a small foreign-body reaction compared with widely used metal electrodes. Capitalizing on the multi-functional gel composite, we fabricated an ultrathin and mechanically flexible organic active matrix amplifier on a 1.2-μm-thick polyethylene-naphthalate (PEN) film to amplify (amplification factor: ∼200) weak biosignals. The composite was integrated to the amplifier, to realize a direct lead epicardial electrocardiography that is easily spread over an uneven heart tissue.

A biocompatible, ultraflexible and thin-film organic amplifier was realized with a biocompatible highly conductive hydrogel-based electrode that comprises a multi-walled CNT sheet and a stretchable elastic aqueous polyrotaxane-based gel with a movable cross-linker (cyclodextrin/polyethylene), which is known as a ‘slide-ring gel' or a ‘topological gel'. The entire CNT surface is uniformly coated with aqueous polyrotaxane-based gel using a hydrophilic ionic liquid. This structure has an increased effective surface ratio (electrode area) and it exhibits an admittance of 100 mS cm^−2^ without sacrificing softness, which is a characteristic of gels. Furthermore, the admittance does not change even in the low-frequency range and its value is two or three orders of magnitude larger than that of conventional gel electrodes. The gel composite is chemically stable and mechanically flexible and stretchable, showing Young's modulus of approximately on the order of 10 kPa. When the gel composite was implanted in the hypodermal tissue of a living body for 4 weeks, the foreign-body reaction of the tissue was smaller than those using existing metal electrodes. Furthermore, by combining the gel composite with an organic-transistor-based mechanically compliant waterproof amplifier manufactured on a 1.2-μm-thick ultraflexible polymeric substrate, we demonstrated a sheet-type ultraflexible electrocardiograph measurement system that can be fully spread over the uneven surface of the heart of a living body. These organic-transistor-based amplifiers have extreme flexibility, whose critical bending radii are much less than 500 μm. The soft and biocompatible gel composite, as well as the amplifiers, enables the sensor system to detect a very small electronic potential; a 1-mV signal coming from a rat heart was amplified by a factor of 200 by the organic amplifiers, thus enabling ultra-sensitivity without any noise related to signal cross-talk and interference.

## Results

### Biocompatible gel electrode

**Figure 1:**
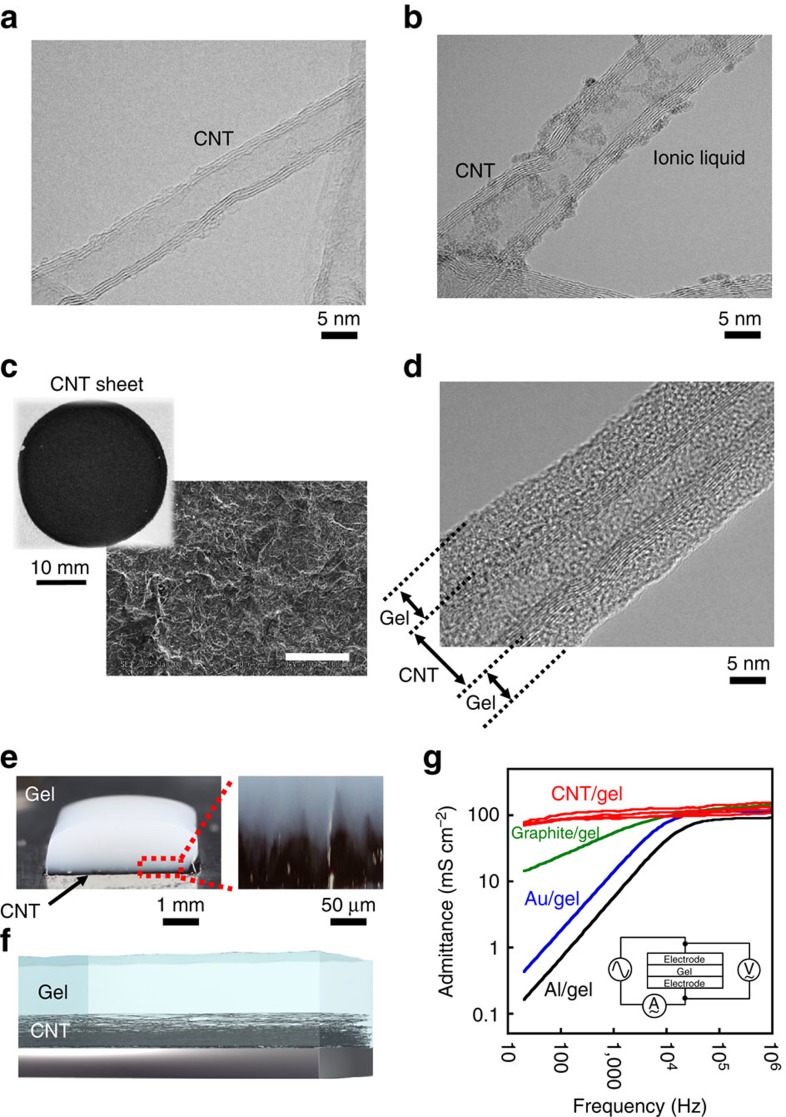
Conductive gel. High-resolution cross-sectional electron microscopy image of (**a**) stand-alone multiwalled CNT and (**b**) ionic-liquid-coated CNT. The specimen was dried in vacuum and imaged by TEM (80 kV). (**c**) Conductive CNT sheet and magnified picture of the surface. The scale of the SEM image is 100 μm. (**d**) High-resolution cross-sectional electron microscopy image of the CNT/polyrotaxane composite. (**e**) Cross-sectional picture of the CNT/polyrotaxane composite comprising a 50- to 100-μm-thick CNT gel layer and a 1-mm-thick polyrotaxane-gel layer. A magnified picture of the CNT/polyrotaxane interface is also shown. (**f**) Schematic cross-section of the conductive gel where a concentration gradient of CNT is formed in the gel. (**g**) Admittance (mS cm^−2^) of CNT/polyrotaxane gel in the vertical direction as a function of frequency, represented as red line. The admittance values of a polyrotaxane gel with different conductive layers are also shown for comparison. Polyrotaxane gel with (red) CNT, (green) graphite sheet, (blue) Au-coated film and (black) Al-coated film. The admittance was derived by subtracting the parasitic resistance in the experimental setup as open/short error compensation.

A biocompatible gel composite is formed by a uniformly dispersed multi-walled CNT composite sheet (CNT sheet) (length: >100 μm and diameter: 5 nm, unless otherwise noted) and a stretchable, soft, elastic and aqueous polyrotaxane-based gel with a movable cross-linker (cyclodextrin/polyethylene)^1^ (Fig. 1 and Supplementary Figs 1–6). Thick CNT bundles (50 mg) were mixed with *N*,*N*-diethyl-*N*-methyl-*N*-(2-methoxyethyl)ammonium tetrafluoroborate (DEMEBF_4_; 100 mg), which is a hydrophilic ionic liquid. Through the above process, the thick CNT bundles were untangled into thin bundles and the CNT surface was then coated with the ionic liquid by self-assembly (Fig. 1b). The resulting suspension with CNTs and ion liquid DEMEBF_4_ was subjected to an automatic grinding system for 6 h to form a black substance, referred to as ‘bucky gel.' The bucky gel (150 mg) was successively added to deionized water (10 ml) and a microfibrillar cellulose (200 mg of water solution containing 10% cellulose, Celish, Daicel Chemical Industries, Ltd, referred to as microcellulose in this study). The mixture was stirred at 25 °C (1 h) and sonicated (UH-50, SMT Co., Ltd) at 30 °C (10 min). The resulting swollen gel was poured onto a polytetrafluoroethylene plate by drop casting and air dried for 24 h to produce a CNT sheet, as shown in Fig. 1c.

A 50- to 150-μm-thick conductive CNT sheet was prepared, followed by a 1-mm-thick polyrotaxane-gel precursor comprising a photo-cross-linking agent (1.0 mg; Irgacure 2959, Nagase & Co., Ltd). In addition, an adamantine-polyrotaxane-based xerogel^1^ (1 g; Advanced Materials Ltd) without CNT was cast to form a bilayer composite comprising a CNT sheet and a polyrotaxane gel. After cross-linking using 365-nm ultraviolet irradiation for 5 min, the bilayer CNT-sheet/gel composite was formed (Fig. 1e,f). This polyrotaxane gel can be easily patterned to form microstructures with a spatial resolution of ∼50 μm via photo-cross-linking using an ultrafine digital ultraviolet exposure system (Supplementary Figs 1–5).

Figure 1a,b,d show the high-resolution cross-sectional electron microscopy images of a single CNT, an ionic-liquid-coated CNT, and a CNT/polyrotaxane gel composite, respectively. The transmission electron microscopy (TEM) (80-kV HF-2000 Cold-FE TEM, Hitachi High-Technologies Corp.) images of the specimen after being dried in vacuum clearly show the polyrotaxane-based gels coated on the multi-walled CNTs with a diameter of 5–10 nm (Fig. 1d). Owing to the hydrophilic ion-based ionic liquids, an aqueous polyrotaxane-based gel (slide-ring gel) was formed around the CNT. Energy-dispersive X-ray (EDX) spectrometry (Genesis APEX2, EDAX Corp.) clearly shows aqueous polyrotaxane-based gels around the CNTs (Supplementary Fig. 6).

As shown in Fig. 1g, the bilayer gel composite had an admittance of 100 mS cm^−2^ even in the low-frequency range, which, to the best of our knowledge, is the highest among CNT-based conductive gels^2^,^3^,^4^. The admittance of the CNT/polyrotaxane gel composite was evaluated through AC measurements, as shown in Fig. 1g. Polyrotaxane-based gels with different conductive layers, such as the graphite sheet, Au-coated film and Al-coated film, are also shown for comparison. The CNT-sheet/gel composite showed the highest admittance in the low-frequency range, owing to the large surface ratio of the CNT-based conductive electrodes, whose admittance was two or three orders of magnitude larger than that of metal/gel-based composite electrodes. In fact, the CNT/gel interface formed a highly capacitive electronic double layer whose capacitance exceeded 140 μF cm^−2^. The CNT-sheet/gel bilayer electrode was chemically stable and mechanically flexible and stretchable, showing Young's modulus below 100 kPa.

### Biocompatible tests

**Figure 2:**
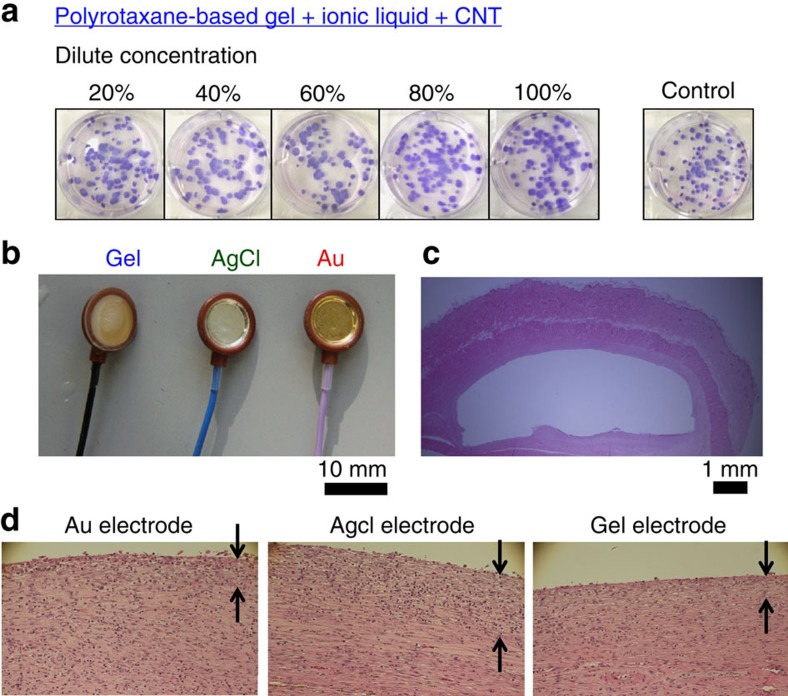
Biocompatibility test 1. (**a**) Colony-forming assay for cytotoxic evaluation. (**b**) Implant assay in living body to evaluate long-term foreign-body response. Three probes that can change the surfaces of the electrodes were used for implantation into the hypodermal tissue of living rats for 4 weeks. (**c**) Pathology graft after explanting the probes and staining. (**d**) Magnified pictures of the surfaces of the pathology grafts. The arrows represent the depth of inflammation reaction.

The biocompatibility was examined through a four-step test. We organized all the biocompatibility tests throughout under a rule described by an internationally standardized procedure, namely colony-forming assay (ISO10993-5) and implant assay (ISO10993-6). First, in a colony-forming assay (ISO10993-5), hamster fibroblasts (V79) were cultivated for cytotoxic evaluation. The conductive gels were finely cut and sterilized by autoclaving (121 °C for 20 min). Next, extraction liquid was extracted at 37 °C for 24 h, which corresponds to an undiluted sample. This liquid was diluted to 20, 40, 60, 80 and 100%. Each diluted liquid was cast on V79 (100 cells per well) and cultivated at 37 °C for 6 days in 5% CO_2_ atmosphere. The number of cells per well was counted after fixing and staining the cells. The number of cells did not change after casting the extraction liquid and the cell state was similar to that of the negative control, which showed that the conductive gels are not cytotoxic (Fig. 2a and Supplementary Figs 7 and 8).

Second, in an implant assay (ISO10993-6), the gel composites were implanted into the hypodermal tissues of living rabbits for 4 weeks. Indeed, 4 weeks is one of the examination periods for ISO10993-6. The degree of foreign-body reaction was quantified based on ISO 10993-6:2007 in terms of the cell type. The irritant ranking score was averaged in four populations, compared them with that of the negative control and classified them into four categories: non-, slight, moderate and severe irritant (Table 1).

Three subcutaneously implanted electrodes whose surfaces were coated with the gel composite, AgCl and Au were used in this experiment, as shown in Fig. 2b and Supplementary Fig. 8. Figure 2c shows the cross-sectional images of a subcutaneous tissue after an electrode was explanted. Figure 2d and Supplementary Fig. 9 show the magnified images of graft pathology after staining. The top surfaces of the grafts were exposed to the three electrodes. Fibrosing cell infiltration was observed near the surface, whose thickness represented the amount of inflammation (indicated as arrows in Fig. 2d). Table 1 lists the implant assays for six samples. The gel composite shows an irritant ranking score of 15.3, which is smaller than that of conventional Au implant electrodes (ranking score: 22). Each irritant ranking score was averaged in four populations and a dominant difference was observed even if we considered the error, indicating that the gels exhibited smaller foreign-body reaction.

**Figure 3:**
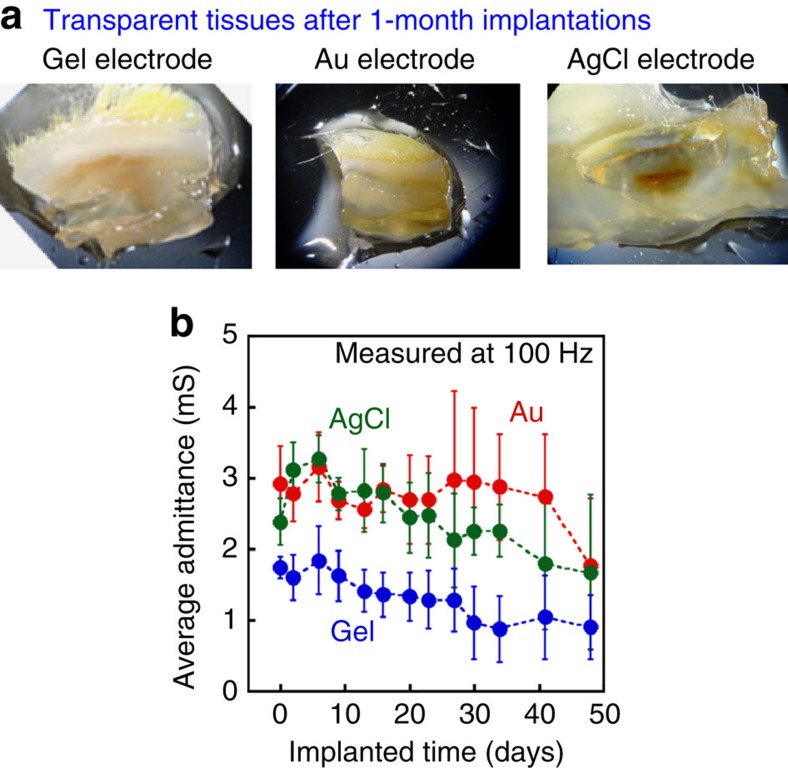
Biocompatibility test 2. (**a**) Samples (pathology grafts) are transferred to the final clearing solution (LUCID: nine parts thiodiethanol and one part glycerol) at room temperature. The cleaned tissues are evaluated using microscopy. Internal haemorrhage is observed in the pathology grafts where the AgCl electrode is explanted. (**b**) Averaged conductance of electrodes subcutaneously implanted in living rats, whose details can be seen in Supplementary Fig. 9.

Third, optical cleaning process was carried out on the above three pathology grafts to evaluate the biocompatibility. Several tissue-cleaning methods have been reported by other groups and used as new analysis techniques for living tissues^5^,^6^,^7^,^8^. The transparent tissues enabled us to observe an internal haemorrhage with inflammation. We found remarkable inflammation from the AgCl electrodes, as shown in Fig. 3a. Therefore, as a result of the laboratory procedure for the three pathology grafts described above, the gel electrode is more compatible with a living tissue.

We have evaluated not only the above three biological assays but also the biocompatibility viewed from the electrical characteristic perspective. The technical details of the result are presented in the Supplementary Information. Figure 3b shows the admittance between each electrode pair through the subcutaneous tissue as a function of the implanted time, where the admittance values were average values obtained from more than three samples. We verified that the gel electrode exhibited stable conductance equal to the conventional metal electrode typically used in biomedical applications, demonstrating the excellent feasibility of the gel electrodes from the viewpoint of electrical performance, mechanical flexibility and biocompatibility.

### Ultraflexible organic amplifier

**Figure 4:**
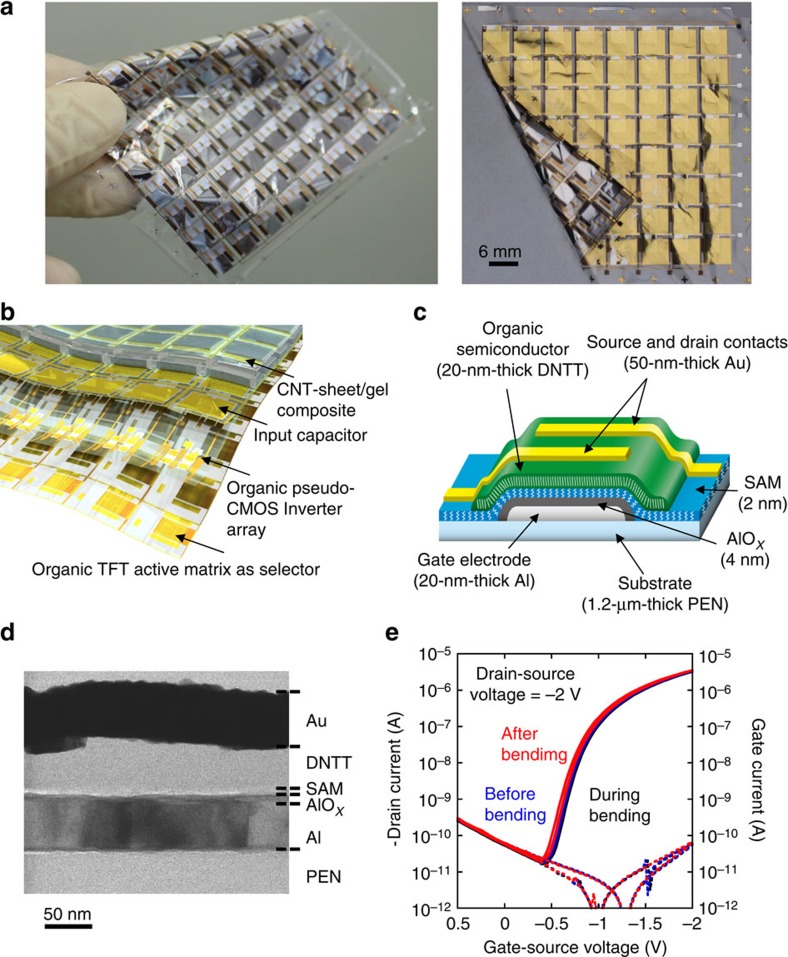
Ultraflexible organic integrated circuits. (**a**) Picture and (**b**) schematic illustration of the 1.2-μm-thick ultraflexible amplifier array comprising organic transistors. (**c**) Cross-sectional image and (**d**) high-resolution cross-sectional electron microscopy image of an organic transistor on a 1.2-μm-thick plastic substrate. (**e**) Electrical characteristics of an organic transistor before, during and after bending to a radius of 50 μm. The transistor is fabricated on a 1.2-μm-thick PEN substrate and 1.3-μm-thick parylene encapsulation stack. The transistor channel is located at the neutral strain position. The transistor characteristics confirm that the devices are not damaged when bent to a radius of 50 μm.

To detect weak biosignals, a two-dimensional array of organic amplifiers was directly fabricated on a 1.2-μm-thick ultraflexible PEN substrate (Fig. 4a). The schematic cross-sectional diagram is shown in Fig. 4b. The amplifier was formed using an inverter with a pseudo-complementary metal-oxide semiconductor (CMOS) layout^9^ comprising four *p*-channel organic transistors with semiconducting dinaphtho[2,3-b:2′,3′-f]thieno[3,2-b]thiophene^10^ and an organic self-assembled monolayer (SAM) gate dielectric^11^,^12^,^13^, a capacitor and a resistor (Supplementary Fig. 13). A waterproof hybrid encapsulation stack comprising a 200-nm-thick Au layer sandwiched between a 100-nm- and 1.2-μm-thick parylene layer was deposited on the transistors to serve as a passivation layer against oxygen diffusion, humidity and mechanical attrition *in vivo*, and as an overcoat layer to enhance mechanical flexibility and durability, because the organic semiconducting layer was located at the neutral strain position^13^, leading to the reduction in the effective strain at the thin-film transistor (TFT) plane. Figure 4c,d show the schematic cross-section and TEM image of one transistor on a 1.2-μm-thick ultraflexible PEN and the manufacturing details and electrical performance are described in the Supplementary Information (Supplementary Figs 12–21). The ultrathin transistors exhibited high mechanical flexibility whose performance did not change even after a 50-μm bending radius, as shown in Fig. 4e and Supplementary Fig. 18.

**Figure 5:**
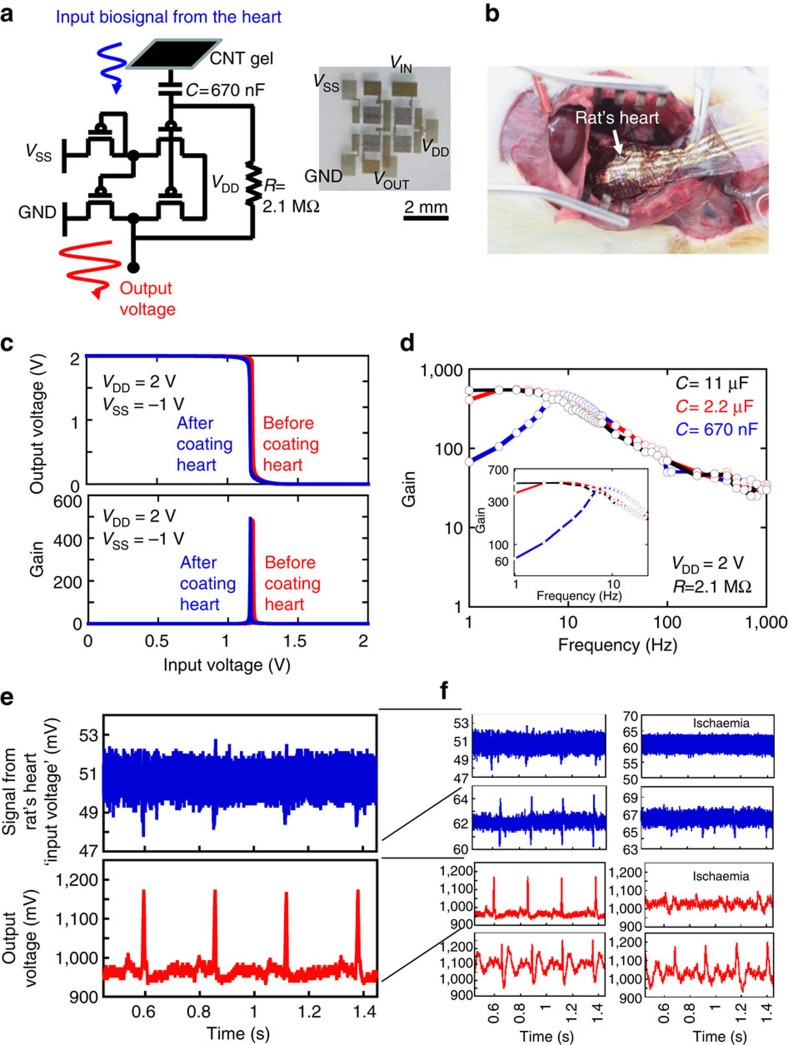
Electrocardiogram using conductive gel probes and ultraflexible organic circuit. (**a**) Circuit diagram of one cell of an organic amplifier with a conductive gel for *in vivo* electrocardiograph comprising an organic pseudo-CMOS inverter that works as an amplifier, where *V*_DD_ is the power source voltage, *V*_SS_ is the tuning voltage, *L* is the channel length and *W* is the channel width. Photograph of an organic pseudo-CMOS inverter is also shown. (**b**) Photograph of ultraflexible circuits (pseudo-CMOS inverter) on the rat heart. (**c**) Characteristics of the pseudo-CMOS inverter before and after coating the rat heart. The electrical performance does not change after coating. (**d**) Frequency responses of the gain of an organic amplifier by varying the input capacitor (**c**) from 0.67 to 2.2, to 11 μF. (**e**). Amplification performance and (**f**) the magnified characteristics of the organic amplifier. (Blue line) Input signal is directly obtained from the heart where CNT conductive gel is used for the electronic interface. (Red line) Output signal is amplified using an organic amplifier. An input signal of 1.2 mV is amplified to a 220-mV output signal. A series of electrocardiograms is also shown in the right. An ischaemia-induced myocardial infarction is clearly observed. The total thickness of the cardiac electrodes is ∼1 mm, while the size is 6 mm × 6 mm, which was determined by the size of a pixel of an organic amplifier (Fig. 5 and Supplementary Fig. 16).

Figure 5a shows the circuit diagram and a photograph of the organic amplifier comprising a pseudo-CMOS inverter on a 1.2-μm-thick ultraflexible PEN with organic circuits, CNT-gel composite (CNT-gel), input capacitor (*C*) and resistance (*R*). The input impedance and power requirements for the amplifiers are 100 kΩ and 10 μW, respectively. The pseudo-CMOS inverter has five electrical terminals: input, output, power source voltage *V*_DD_, tuning voltage *V*_SS_ and ground (GND). It can operate within 2 V with a signal gain exceeding 400 (Fig. 5c and Supplementary Fig. 21) and it can maintain this electrical performance even when bent at a bending radius of 50 μm. The propagation delay per stage of the transistors was 23.4 μs at 2 V, which corresponds to the frequency response of 42.7 kHz on a single transistor, as shown in the Supplementary Information (Supplementary Fig. 20)^9^. The excellent electrical and mechanical performance of the inverter was demonstrated by evaluating its characteristics before and after being used to coat a rat heart; the change in the characteristics was negligible (<1%) (Fig. 5b,c).

Figure 5d shows the amplifier gain obtained from the organic amplifier for this experiment. The frequency responses of the gain were similar at frequencies exceeding 10 Hz. However, the amplifier with larger capacitance (*C*) showed higher gain even below 10 Hz. This result indicates that the input capacitor should be carefully chosen in accordance with the required monitoring frequency.

### Electrocardiogram monitoring

Next, a direct epicardial electrocardiogram was obtained using self-feedback-type organic amplifiers by combining the inverter, a conductive gel sheet, a resistor made of conductive carbon paste (*R*=2.1 MΩ) and a capacitor formed using SAM/AlO_*x*_ dielectrics sandwiched by Au electrodes (*C*=670 nF) (Fig. 5a and Supplementary Fig. 13). The gel composite was placed on the rat heart and interconnected to an organic amplifier sheet using electrical wirings. A 1.2-mV input signal obtained from the heart was amplified to a 220-mV output signal using the amplifier (signal gain: ∼200) (Fig. 5e,f). Here we note that a high amplifier gain exceeding 100 was obtained, owing to a large capacitor of 670 nF and an internal resistance of 10–50 kΩ, whose gain is consistent with the theoretical estimation. The input SNR of 0.53 was increased to 64 after amplification, which is the first demonstration of amplification of *in vivo* biosignals from a hypodermal tissue using organic circuits. The amplifier gain exceeded 10 at below 1 kHz and 100 at below 100 Hz (Supplementary Fig. 21). The frequency dependence did not affect the measurement accuracy and the amplifier gain was sufficiently large, considering that important heart signals lie within 1 kHz (ref. 14). The low-frequency gain can be increased by optimizing the passive components (resistance and capacitance) of the organic amplifier, as shown in Fig. 5d.

## Discussion

One of the ultimate goals of *in vivo* monitoring systems is to elucidate biological activities at the organ level, such as in the brain and in the heart, with high spatial and temporal resolution over a large area. From this viewpoint, flexible electronic devices^13^,^15^,^16^,^17^,^18^ have been intensively studied, because they can be applied on complex curved surfaces with a large area coverage. In recent times, flexible *in vivo* neural interfaces have been demonstrated using silicon nanomembrane TFT active matrices to electrically monitor neural activities in a cat brain with high spatial and temporal resolution^18^, and to investigate the mapping of neural circuits in acute brain slices^19^. Organic TFTs^20^,^21^,^22^ and related structures^23^,^24^,^25^ have been investigated to further improve mechanical flexibility.

Long-term stability and reliability are the biggest challenges for *in vivo* electronic systems. Realizing long-term *in vivo* monitoring is very difficult, because monitoring biosignals requires sensitivity on the order of microvolts to millivolts. Furthermore, the inherently wet and deformable surfaces of biological tissues result in further difficulty in monitoring very small biosignals. We have demonstrated fabrication of smart stress-absorbing electronic devices that can adhere to wet and complex tissue surfaces for reliable and long-term electronic measurements of vital signals^26^. Combining the adhesive gel technology with the biocompatible active amplifier proposed in the work, more precise measurement can be realized *in vivo* for a long time.

Highly conformable conducting poly(3,4-ethylenedioxythiophene) poly(styrenesulfonate) electrodes have been reported for *in vivo* electrocorticography^27^,^28^. Au-coated poly(dimethylsiloxane) has been demonstrated for stretchable *in vivo* neural interfaces^29^. However, the biocompatibility of these soft electrodes in contact with the tissues for a long duration has not yet been evaluated. Furthermore, an electronic system combined with soft electrodes has not yet been demonstrated. Replacing conventional metal electrodes with soft conducting materials is a promising solution to obtain reliable large-area mechanical and electrical contacts at the bio/electrode interfaces. Moreover, integration of soft conductive gels with ultraflexible electronic amplifier is indispensable in realizing *in vivo* monitoring periods with sensitivity on the order of microvolts to millivolts.

Flexible silicon nanomembrane transistors have been manufactured for buffering biological signals and multiplexing electrodes to record the spatial properties of a cat brain activity *in vivo*, demonstrating the excellent feasibility of flexible electronic systems^18^. In our work, an organic-based transistor with an ultraflexible PEN substrate, instead of inorganic-based transistors with silicon membranes, was found to improve mechanical flexibility. Furthermore, the use of a biocompatible conductive gel as an electrode enabled long-term sustainable *in vivo* circuits. The use of organic amplifiers near a living tissue enabled high-resolution, high-sensitivity and multi-channel monitoring with improved SNR.

A previous report regarding organic-transistor-based implantable electronic systems was presented by Feili *et al.*^30^, who manufactured a stimulation array to a heart using organic-transistor active matrices. Moreover, the cytotoxicity of the transistors was evaluated with respect to the potential effects on cell viability^30^. Huang *et al.*^31^ have demonstrated implantable electrocardiogram monitors using single-probe ultrasonic apparatus, which had already been actually used in clinical practices^32^. However, the novelty of our works lies in the complete system of an ultrathin electronic amplifier sheet with ultrasoft and biocompatible gel. To the best of our knowledge, this technique is the first integration of an ultrathin active matrix amplifier array with biocompatible gels and the first demonstration of electrocardiogram measurement of a heart of a living rat. Furthermore, we have successfully monitored ischaemia-induced myocardial infarction on a living heart, which was amplified using the ultrathin organic amplifier. Owing to the ultraflexible, ultrathin and ultralightweight thin-film amplifier, it can conform to dynamic motion such as that in a living heart. It can reduce the burden of long-term monitoring. Furthermore, we will no longer be concerned of the lifetime of organic circuits.

In conclusion, by using novel gelatinous composite as a biocompatible electrode for implantable electronics, we have succeeded in reducing foreign-body reaction compared with the widely used metal electrodes, whereas the new material maintains extraordinarily high AC admittance of 100 mS cm^−2^ even in the low-frequency range. Intensely viewed from multiple perspectives, this gel composite was quantitatively evaluated by four different types of biocompatibility tests. Both standard *in vitro* and *in vivo* biocompatibility tests, namely colony-forming assay (ISO10993-5) and implant assay (ISO10993-6) using hypodermal tissues of living rabbits for 4 weeks, clearly demonstrated that the implantable gel composite electrodes show minor foreign-body reaction compared with the widely used metal electrodes. This result is also consistent with the *in vivo* impedance measurements of our gel electrode implanted for 48 days. Furthermore, one of the highlights in the manuscript is that the newly developed ‘tissue-cleaning method' was used for the first time, to the best of our knowledge, to characterize biocompatibility. The new three-dimensional imaging using the tissue-cleaning method has unambiguously established that no internal haemorrhage with inflammation was observed using the gel electrode, whereas the AgCl and other metal electrodes exhibited remarkable inflammation. This study is a very important step towards realizing long-term implantable monitoring systems.

Furthermore, the abovementioned gel composites were integrated with a two-dimensional array of ultrathin, ultraflexible organic amplifiers to make the interfaces between the bio tissues and electrodes of the electronics biocompatible. The world's thinnest ultrathin organic amplifier system was directly laminated over a complex and dynamically moving surface of a living heart, while minimizing the mechanical interference due to motions. The feasibility of the system is demonstrated by the direct measurement of epicardial electrocardiogram signals at an amplification factor of 200, which, to the best of our knowledge, is the largest among flexible amplifier circuits. Furthermore, we have successfully monitored ischaemia-induced myocardial infarction on a living heart. The combination of biocompatible gel composites and ultrathin organic electronics can reduce the burden of long-term monitoring of bio-signals and, therefore, it will broaden the potential application of flexible biomedical electronics from disposable flexible electronics used only during medical surgery to long-term implantable monitoring systems.

## Methods

### Approval for animal testing

The protocols for the animal experiments were approved by the Institutional Animal Care and Use Committee of the University of Tokyo (Approval numbers: KA12-1 and P08-020). The cytotoxic evaluation of gel composite was performed based on a colony-forming assay using hamster fibroblasts (V79, unauthenticated) obtained from the Health Science Research Resources Bank, Japan Health Sciences Foundation. Mycoplasma contamination was not detected in an inspection using a dedicated test kit (MP Biomedicals, LLC). The implant assay of gel composites was performed using male JW/csk rabbits whose weights ranged from 2.9 to 3.6 kg. These colony-forming assay and implant assay were carried out by Kamakura Techno-science Co., Ltd, Japan, according to the internationally standardized procedures ISO10993-5 and ISO10993-6. The evaluation of gel electrode characteristics and the recordings of electrocardiogram were carried out using 12-week-old male Wistar rats. The implant tolerance test of organic transistors was performed using a 4-year-old female Saanen goat.

### Water dispersibility of CNTs

The water dispersibility of five CNT samples was tested—all samples were first stirred in deionized water at 25 °C using a magnetic stirrer (>700 r.p.m.) for a week and pictures were taken after the stirring was stopped—(1) CNT (30 mg); (2) mixture of CNT (30 mg) and DEMEBF_4_ (60 mg); (3) CNT (30 mg) subsequently processed on a high-pressure jet-milling homogenizer (60 MPa; Nano-jet Pal, JN10, Jokoh); (4) mixture of CNT (30 mg) and DEMEBF_4_ (60 mg) subsequently processed on the homogenizer; and (5) mixture of CNT (30 mg), DEMEBF_4_ (60 mg) and microfibrillar cellulose (100 mg water solution containing 10% cellulose; Celish, Daicel Chemical Industries, Ltd) subsequently processed on the homogenizer. Each sample was then photographed (see Supplementary Fig. 1A). Samples (4) and (5) showed excellent dispersibility of CNT in water. High-resolution scanning electron microscope (SEM) images of Samples (1–4) were collected (Supplementary Fig. 1B) showing the dispersibility of CNTs. The specimens were dried in air before the SEM observation. The high-pressure jet-milling homogenizer can effectively untangle the CNT bundles, as observed in Sample (3).

### Manufacturing process of CNT composite sheet

Supplementary Fig. 2A shows the schematic of the fabrication process. Fabrication of the conductive CNT sheet was realized, because the ultrafine bundles of the CNT could be uniformly distributed in microfibrillar cellulose using room-temperature, aliphatic-system hydrophilic ion-based ionic liquids. CNTs (with purity of >99.98%) were used as highly conductive and chemically stable dopant. The CNTs (50 mg) were mixed with 100 mg of hydrophilic ionic liquid DEMEBF_4_ and the resulting suspension was subjected to automatic grinding for 1 h to form a black substance, referred to as ‘bucky gel.' The gel (150 mg) was successively added to deionized water (10 ml) and processed on a high-pressure jet-milling homogenizer (60 MPa; Nano-jet Pal, JN10, Jokoh). The mixture was stirred at 25 °C (1 h) and added to microfibrillar cellulose (200 mg of water solution containing 10% cellulose, Celish, Daicel Chemical Industries, Ltd, referred to as microcellulose in this study). The resulting swollen gel was poured onto a polytetrafluoroethylene plate by drop casting and air-dried for 24 h to produce a CNT sheet. The fabricated CNT sheet had a surface with highly uniform entanglement between the CNT and cellulose, resulting in a large surface ratio (large capacitance when dielectric materials are deposited on the surface). Supplementary Fig. 2B shows the SEM images of the CNT sheet as a function of the cellulose content. With the increase in the cellulose content, the surface became rough.

Supplementary Fig. 3A shows that the conductivity of the conductive CNT sheet strongly depends on the cellulose content. In the CNT sheet, the cellulose content was changed from 5  to 65 wt%, whereas the amounts of CNT and DEMEBF_4_ were 50 and 100 mg, respectively. When the cellulose content was >40 wt%, the CNT sheet was thick and porous; when it was <10 wt%, the CNT sheet became porous and fragile. We found that the highest conductivity could be obtained when the cellulose content was ∼12 wt% and the mixing ratio of CNT and DEMEBF_4_ was 1:2. The CNT easily formed strongly entangled bundles because of the strong covalent bonds, thus resulting in a well-developed entanglement from an unfavourable re-aggregation in the polymer matrix or other base materials. However, ionic liquids can prevent the entanglement of CNT. The high conductivity of the sheet is mainly due to the uniform dispersion of CNT as conductive dopants into the cellulose using ionic liquids.

Supplementary Fig. 3B shows that the conductive paper looks and feels like a regular paper. Moreover, it is used as wiring to transmit power from a battery to a light-emitting diode, demonstrating the very high conductivity of the CNT sheet (Supplementary Fig. 3C). The AC admittance of the CNT/gel electrodes can be observed in Supplementary Fig. 3, obtained from four different samples. The magnified image is also shown in the inset.

### Another manufacturing process of CNT/gel composite

Using the above-described water-dispersed CNTs, a CNT/gel composite was fabricated (see Supplementary Figs 4 and 5). The CNTs were swollen and uniformly dispersed in water by stirring with a hydrophilic ionic liquid followed by homogenization by high-pressure jet milling. The resulting paste containing dispersed CNT was easily mixed with a photo-cross-linking agent (Irgacure 2959, Nagase & Co., Ltd) and an aqueous cyclodextrin/polyethylene-based polyrotaxane (Advanced Materials Ltd) to form an aqueous CNT/polyrotaxane composite, which is a precursor of CNT conductive gels. The composite was then cast onto a glass plate and was covered with a glass plate. The thickness was controlled using a spacing sheet (50 μm in this experiment). Next, 365-nm ultraviolet light was irradiated on the composite to obtain a 50-μm-thick CNT conductive gel sheet. The swelling ratio is ∼2,000 and the volume content of water is 90% in the gel/composite.

A digital ultraviolet exposure system (PMT Corporation, Japan) can create a very fine ultraviolet source with a linewidth of <1 μm. In this experiment, we used a linewidth of 50 μm. The minimum linewidth of the gel depends not only on the spot size of the ultraviolet exposure but also on the thickness of the gel and its adhesive characteristic to the substrates.

The gel/CNT sheet containing microfibrillar cellulose has an adhesive characteristic and easily adheres to the electrodes of an active matrix amplifier. Furthermore, our gel layer can be patterned using a photolithographic process. After the fabrication of the active matrix amplifier, it is coated with the gel precursor and then exposed to light, to pattern the gel on the electrodes of the amplifier.

Young's modulus of the composite gel is ∼10 kPa, which is almost the same as that of a brain (the softest region in living creatures), which means that a softness of 10 kPa or less is good enough for electrodes to measure biological signals. Young's modulus was measured using conventional compression and resonance methods.

### TEM observation of CNT/rotaxane composite

High-resolution cross-sectional electron microscopy images of three samples were obtained, as shown in Supplementary Fig. 6: (A) multi-walled CNT, (B) CNT/polyrotaxane composite without ionic liquid ((rotaxane (100 mg) and jet-milled CNT (30 mg) mixture was stirred in water) and (C) CNT/polyrotaxane composite with ionic liquid (as described in the main text). The specimens were dried in air and imaged by TEM (80-kV HF-2000 Cold-FE TEM, Hitachi High-Technologies Corp.). Sample A showed a multi-walled CNT structure with three to four walls. Sample B showed a multi-walled CNT structure and a heterogeneous incrustation on the surface, which could be a rotaxane. Sample C showed a very uniform incrustation on the CNT surface. We believe that DEMEBF_4_ coated the CNT surface owing to its large surface energy and then promoted adhesion of hydrophilic polyrotaxane. Elementary characterization by EDX spectrometry is also shown. Aqueous polyrotaxane-based gels were detected around the CNT, as shown in the figure.

### Colony-forming assay for cytotoxic evaluation

Supplementary Fig. 7A shows the procedure of the colony-forming assay with results summarized in Supplementary Table 1. This assay was performed in a certified independent evaluation organization with the Good Laboratory Practice (Kamakura Techno-science Co., Ltd.) according to the guidelines for preclinical biological evaluation of medical materials and devices (Ministry of Health, Labour and Welfare, Japan, memorandum, JIMURENRAKU Iryokiki-Shinsa, 36, 2003 and ISO 10993-5:2009: the Biological Evaluation of Medical Devices—Part 5: Tests for *In Vitro* Cytotoxicity).

We cultivated 100 Chinese hamster fibroblasts (V79) in a well seeding. First, the CNT/gel composites were finely cut and sterilized by autoclaving (121 °C for 20 min). Then, the extraction liquid was extracted using extraction culture media (non-essential amine acid containing minimum essential medium with 5% fetal bovine serum: MO5) at 37 °C for 24 h, which corresponds to an undiluted sample, with an extraction ratio of 0.1 g ml^−1^. The extraction liquid was diluted to 20, 40, 60, 80 and 100%. Each diluted liquid was cast on V79 (100 cells per well) and cultivated at 37 °C for 6 days in 5% CO_2_ atmosphere. Next, the cells were fixed using methanol for 15 min and dyed using 5% Giemsa stain for 15 min, and the number of cells was counted. A negative control (high-density polyethylene sheet), positive control A with moderate cytotoxicity (0.1% zinc diethyldithiocarbamate (ZDBC)-containing polyurethane film) and positive control B with mild cytotoxicity (0.25% ZDBC-containing polyurethane film) were processed to guarantee the extraction operation. An additional positive control (ZDBC) was also processed to guarantee the sensitivity of the V79 cells.

Supplementary Fig. 7B shows the results of the assay. The polyrotaxane-based gel and CNT/polyrotaxane gel composite without ionic liquid showed no changes in the number of colonies even after the extraction liquid was cast and the cell state was similar to that of the negative control. The CNT/polyrotaxane gel composite with ionic liquid also showed no changes. These results suggest that the CNT conductive gels are not cytotoxic. Supplementary Fig. 8 shows additional details about the counting of the number of cells and the results.

### Implant assay for biocompatibility evaluation

The assay was performed in a certified independent evaluation organization in Good Laboratory Practice (Kamakura Techno-science Co., Ltd) according to ISO 10993-6:2007: Biological Evaluation of Medical Devices—Part 6: Tests for Local Effects After Implantation.

For this experiment, three types of bioprobes were prepared, as shown in Supplementary Fig. 8A. The surfaces of the electrodes in these probes were coated with Ag/AgCl (commercially available) (Type1), Ag/AgCl/Au (50 nm) (Type2) and Ag/AgCl/Au/Gel (Type3). The detailed structure of Type3 can be seen in the right figure, which is indispensable for fixing the gel to the electrode. These three electrode types were implanted into the hypodermal tissues of living rabbits for 34 days. The position of the electrodes and the population for the implantation test are shown in Supplementary Fig. 8B. We measured the impedance between the electrodes at daily intervals using an LCR meter (E4980, Agilent), whose current is <0.1 mA, to avoid inflammation by continuous impedance measurements. The average impedance was calculated in terms of the admittance, as shown in Fig. 3b.

Supplementary Fig. 9 shows the magnified cross-sectional images of the raft pathology by staining a subcutaneous tissue after an electrode was explanted (this is the same experiment shown in Fig. 2b–d, for reference). The top surfaces of the grafts were exposed to the three electrodes. Fibrosing cell infiltration was observed near the surface.

### Reproductive experiment under different conditions

CNT conductive gels (size: 1 × 1 cm^2^) were implanted into the hypodermal tissues of living rabbits for 1 and 4 weeks. Next, pathology grafts with samples were dyed and analysed by a certified pathological specialist. The degree of foreign-body reaction was quantified based on ISO 10993-6:2007 in terms of the cell type (polymorphonuclear cells, lymphocytes, plasma cells, macrophages and giant cells), necrosis and response (neovascularization, fibrosis and fatty infiltrate). We averaged the irritant ranking scores of the four populations, compared them with those of the negative control and classified them into four categories: non-, slight, moderate and severe irritants.

Supplementary Fig. 10 shows the graft pathologies of the four different samples after implantation for 4 weeks: (A) high-density polyethylene sheet as negative control sample, (B) 200-nm-thick Au layer on 75-μm-thick polyimide substrate as a conventional metal electrode, (C) polyrotaxane-based gel with ionic liquid and (D) CNT/polyrotaxane gel composite with ionic liquid. All graft pathologies, except for the negative control sample, showed fibrosing cell infiltration at the sample interface. Here, the Au electrode caused much larger cell infiltration than the gel-based materials. Supplementary Tables 2 and 3 show more quantitative data analysed from the pathology grafts and a summary of the results. The results clearly indicate the good biocompatibility of the CNT/polyrotaxane gel composite.

### Tissue-cleaning method

After explanting the above (Type1), (Type2) and (Type3) electrodes, the animals were transcardially perfused with PBS followed by 4% paraformaldehyde (Sigma-Aldrich, St Louis, MO) in PBS. To label the blood vessel wall, Texas Red-labelled lectin from Lycopersicon esculentum (700 μg lectin for rats, Vector Laboratories, Inc.) was intravenously administered before the perfusion fixation. Hypodermal tissues with implanted gels were postfixed in paraformaldehyde at 4 °C for 24 h. To optically clean the tissues, the tissue blocks were washed in water for 3–16 h at 4 °C and incubated in pretreatment solution 1 (two parts thiodiethanol, four parts glycerol and four parts 30% sucrose solution) for 24 h at room temperature and solution 2 (five parts thiodiethanol and five parts glycerol) for 24 h at room temperature. The samples were then transferred to the final clearing solution (LUCID: nine parts thiodiethanol and one part glycerol) at room temperature. For accurate adjustment of the refractive index of the solutions, the samples were moved to a fresh LUCID solution the next day and were stored at 4 °C until the scheduled multi-photon microscopic observation. All washes and incubations were done in a light-resistant container by constant and gentle shaking. Two days after immersion in LUCID, a satisfactory cleaning effect was achieved. For multiphoton microscopy, excitation was achieved using Chameleon Vision II with a laser oscillator at 860 nm (Coherent, Santa Clara, CA) or Spectra-Physics MaiTai DeepSee with a laser oscillator at 860 nm (Newport, Santa Clara, CA). A Nikon A1RMP-only Ti GaAsP two-photon microscope (Nikon, Tokyo, Japan) was used (step size, 3 μm) with objective lenses Nikon CFI LWD × 16 (numerical aperture, 0.8; working distance, 3 mm) and Nikon CFI75 APO 25 × W MP (numerical aperture, 1.1; working distance, 2 mm). The images were processed using NIS-Elements C and AR (Nikon). Tiled images were obtained with 20% overlap. Second-harmonic generation was obtained at 436 nm/20 nm (central wavelength/bandwidth). Texas Red and DyLight595 fluorescence were obtained at 629 nm/53 nm (the images are shown in Supplementary Fig. 11).

### Organic transistors in ultrathin polymeric substrates

We manufactured a two-dimensional array of organic amplifiers comprising four organic transistors on a 1.2-μm-thick PEN substrate (Fig. 4). Transistor gate electrodes were prepared on the surface of a 1-μm-thick PEN substrate by evaporating a 30-nm-thick Al layer through a shadow mask. A 4-nm-thick (AlO_*x*_) layer was then formed on the Al surface by oxygen plasma treatment (5 min, 300 W). The substrate was then immersed in a 2-propanol solution of n-octadecylphosphonic acid, to create a densely packed 2-nm-thick organic SAM on the oxidized Al surface. The total dielectric gate thickness was therefore 6 nm and it had a capacitance per unit area of 0.6–0.65 μF cm^−2^. Some 50-nm-thick layers of dinaphtho[2,3-b:2′,3′-f]thieno[3,2-b]thiophene (DNTT), an organic semiconductor, for the *p*-channel TFTs were then deposited by vacuum sublimation through shadow masks. The source and drain contacts were prepared on top of the organic semiconductors by evaporating Au to a thickness of 50 nm through a shadow mask.

### *In vivo* implant tolerance of organic transistors

Organic transistors must show *in vivo* implant tolerance, in addition to biocompatibility. The encapsulation stack comprised 100-nm-thick parylene (diX-SR, Daisankasei Co., Ltd), 200-nm-thick Au and 1.2-μm-thick parylene layers on organic transistors. The entire organic transistor sheet was coated with polyrotaxane-based gel, immersed in saline and then sterilized by autoclaving at 121 °C for 20 min. The technical details of encapsulation and thermal resistance of the organic transistors can be seen in refs 33, 34. The electrical mobility changed by 3.2% and the threshold voltage shift was −0.35 V after high-temperature sterilization (Supplementary Fig. 12B). The sterilized organic transistor sheet was implanted into a hypodermal tissue of a goat (Supplementary Fig. 12A). Although the off-currents of the transistors were one order of magnitude larger than those before implantation, the on/off ratio exceeded 10^4^. Furthermore, the average mobility changed by <4.3% and the average threshold voltage (*V*_th_) was −0.13 V (Supplementary Fig. 12C). This result indicates that the organic transistors maintain their electronic performance owing to their excellent encapsulation, enabling the design of sophisticated *in vivo* integrated circuits.

### Integration methods for 1.2-μm-thick organic circuits

Supplementary Fig. 13A shows an array of ultraflexible organic pseudo-CMOS inverters on a 1.2-μm-thick PEN substrate. These circuits comprised an ultrathin substrate, enabling them to be spread over arbitrary curved surfaces (Supplementary Fig. 13B). The ultraflexible amplifier comprised the pseudo-CMOS inverter with a transistor active matrix, capacitors and resistors. The contact holes of each layer were produced using CO_2_ laser and/or green laser, depending on the materials and the size required for contact holes. The matrix, manufactured at the bottom of the pseudo-CMOS inverter substrate, was used to address the pixel position. Supplementary Fig. 13C shows the circuit diagram of the active matrix amplifier array. The active matrix amplifier system comprising organic transistors (selector TFT) enables random-access readout with high spatiotemporal resolution and sensitivity. Supplementary Fig. 13D shows a pseudo-CMOS inverter comprising four *p*-type transistors. The channel widths and lengths of the transistors were precisely designed to detect and amplify weak biosignals. *M*_1_ has a channel width and length of 2,000 and 20 μm or 1,000 and 10 μm, respectively, whereas *M*_2_, *M*_UP_ and *M*_DP_ have a channel width and length of 6,000 and 20 μm or 3,000 and 10 μm, respectively. An input capacitor array is also shown.

The conductive CNT-sheet/gel composites were manually integrated to an ultrathin-film amplifier array. The gel/CNT-sheet composite containing microfibrillar cellulose has an adhesive characteristic and easily adheres to the electrodes of the active matrix amplifier, although it strongly depends on the degree of the content of the microfibrillar cellulose. Furthermore, our gel layer can be patterned using a photolithographic process. After the fabrication of the active matrix amplifier, the gel composites are placed on the input electrodes of the amplifier array. Owing to the parylene encapsulation layers, active matrix amplifier and the input electrodes maintain their electrical performance *in vivo*. These layers, manufactured independently, were laminated and integrated using anisotropic conductive films (ANISOLM, Hitachi Chemical Co., Ltd).

Material profiling and cross-sectional imaging of the organic transistors on a 1.2-μm-thick substrate was performed using ultrahigh resolution scanning TEM (STEM) and EDX analysis system. Supplementary Fig. 14A shows a cross-sectional image of the organic transistors on a 1.2-μm-thick substrate using ultrahigh resolution STEM (200-kV HD-2700 Cs-corrected STEM, Hitachi High-Technologies Corp.). The cross-section shows four layers. Supplementary Fig. 14B shows the characterization by EDX spectrometry. The spectra show that only a few nanoscale metal and molecule structures can be formed on very thin plastic substrates. Supplementary Fig. 15 shows other cross-sectional images and profiles of the constituent materials of the organic transistors. These spectra validate the feasibility of our manufacturing process.

### Morphology of DNTT on SAM

The morphology of DNTT was determined by atomic force microscopy. We prepared two different samples: DNTT manufactured on SAM dielectric gate with SiO_2_ wafer and that with a 1.2-μm-thick PEN substrate; the other layers were identical. The average mobility of DNTT on the respective surfaces was 2.3 and 1.0 cm^2^ V^−1^ s^−1^. The lower mobility on the PEN substrate might be due to the slightly rough surface of the SAM dielectric gate layer affected by the substrate roughness. The smoothness of the respective surfaces was ∼0.18 and 2–3 nm RMS. The grain size of DNTT on the PEN substrate was slightly smaller than that on the SiO_2_ wafer, which might also be due to the substrate roughness (Supplementary Fig. 16).

### Manufacturing process on a 1-μm-thick substrate

Supplementary Fig. 17A shows the drain current as a function of the drain-source voltage of a DNTT transistor (*V*_GS_ in steps of 0.5 V). Supplementary Fig. 17B shows the drain and gate current as a function of the gate-source voltage for a DNTT transistor (*V*_DS_: −2 V). The transfer characteristics of the ten transistors were plotted to show the variation. Because of the small thickness of the gate dielectric (6 nm), the operating voltage was ∼2 V. The average field-effect mobility was 1 cm^2^ V^−1^ s^−1^ from the transfer characteristics. The channel width and length of the transistors were 500 and 50 μm, respectively.

The manufacturing process, especially the oxygen plasma treatment, affects the electrical performance of organic transistors. Supplementary Fig. 17C shows the mobility, on/off ratio and saturation current of four different organic transistors under various plasma process conditions: (A) 100 W for 10 min, (B) 150 W for 5 min, (C) 150 W for 10 min, (D) 300 W for 5 min and (E) 300 W for 10 min; the other manufacturing conditions were fixed. Condition (D) produces the best mobility of 1 cm V^−1^ s^−1^ and large on/off ratio exceeding 10^5^. The other conditions result in lower mobilities mainly because of the rougher surface in condition (E) and the lower packing density of the SAMs on the AlO_*x*_ layer due to insufficient plasma exposure.

### Bending test

Supplementary Fig. 18 shows the experimental setup for the bending test of the organic transistors. Bending stresses were applied to the flexible circuits fixed on the stage and push plate using a numerically controlled mechanical stage. The bending radii were precisely measured using a digital microscope (Keyence) from the side of the flexible circuits. All measurements were performed in ambient air. The transistor characteristics were measured using a semiconductor parameter analyser (B1500A, Agilent) with manual probes.

### Design of organic transistor for higher amplifier gain

To obtain high amplifier gain and large power to amplify weak biosignals, the dimensions of the four organic transistors, especially the channel width (*W*) and length (*L*), should be carefully designed. Typically, we design organic transistors with *W*=6,000 μm and *L*=20 μm, which can produce large currents exceeding 100 μA and leakage current on the order of nanoamperes, owing to the excellent insulating characteristics of the SAM dielectric gate layer (Supplementary Fig. 19). The mobility was 0.9 cm V^−1^ s^−1^. Depending on the frequency response required for the applications, we also designed organic transistors with *W*=3,000 μm and *L*=10 μm, which is much faster than those with *W*=6,000 μm and *L*=20 μm, as described below.

### Pseudo-CMOS inverter

Although the pseudo-CMOS inverter comprises four *p*-type transistors (Supplementary Fig. 20A), the inverter characteristics are superior to those of the organic unipolar inverters and conventional organic CMOS inverters. Supplementary Fig. 20B shows the output voltage and gain as a function of the input voltage, where *V*_SS_ is −1 V and *V*_DD_ varies from 2 to 0.5 V. This inverter can operate within 2 V and the signal gain exceeds 400 even at *V*_DD_=0.5 V. Thus, excellent inverter characteristics are exhibited even at a 2-V operation.

Supplementary Fig. 20C shows the oscillation frequency of a five-stage ring oscillator comprising five pseudo-CMOS inverters, which was cited in our previous report^9^. From the result, the operating speed of the single transistor was 42.7 kHz and the propagation delay per stage was 23.4 μs. This temporal resolution is sufficiently fast; thus, the system can measure the biological signals, which are within 1 kHz.

### Signal gain and frequency response of organic amplifiers

Supplementary Fig. 21A shows the circuit diagram of an organic amplifier with a CNT gel. Supplementary Fig. 21B shows the output signal voltage and amplifier gain as a function of the input signal voltage. The former is proportional to the latter and the gain is almost constant (∼100 at input exceeding 2 mV). Here we should note that the high amplifier gain exceeding 100 was obtained, owing to the large capacitor of 670 nF and internal resistance of 10–50 kΩ, whose gain is consistent with the theoretical estimation indicated below.

Supplementary Fig. 21C shows the frequency response of the amplifier gain. The same frequency response was obtained for repeated experiments, demonstrating excellent reproducibility. The frequency response of the amplifier gain strongly depends on the input capacitor (*C*). To evaluate the effect of *C*, we constructed three different amplifiers with *C* values of 0.67, 2.2 and 11 μF.

The spatial resolution of the amplifier array was determined by the input capacitance of the amplifier, whose size was 6 mm × 6 mm. The periodicity of the circuit was 7 mm. The temporal resolution was determined by two operating speeds: one was the operating speed of the amplifier, which corresponded to the recording speed; the other was the operating speed of the pseudo-CMOS inverter in the amplifier, which corresponded to the addressing speed in the active matrix amplifier. Figure 5d in the main text and in Supplementary Figs 20C and 21C show the operating speed of the amplifier (recording time) whose amplifier gains were 550 in the frequency range from 1 to 30 Hz, 71 at 100 Hz and 30.9 at 1 kHz.

### Detection of ischaemic state due to myocardial infarction

Supplementary Fig. 22 shows a picture of the ischaemic state of a rat heart due to myocardial infarction. A coronary artery was ligated to induce a myocardial infarction in the right half of the heart. Figure 5f shows the input signal voltage of the abnormal rat heart and the amplified output signal voltage from the organic amplifier.

### Effects of the CNT-sheet/gel-composite electrode

To evaluate the conductive-gel sheet, body surface electrocardiographs were obtained using two different gel probes (size: 1 × 1 cm^2^, thickness: 1 mm) for comparison. One is a commercially available conductive gel comprising a graphite-sheet electrode with acrylamide gel (Supplementary Fig. 23A) and the other is a sheet (Supplementary Fig. 23B). In this experiment, we used a conventional amplifier system marketed for medical use (Neuropack μ, MEB-9104; Nihon Kohden Co., Ltd, Tokyo, Japan; gain: 3,000) to evaluate the stand-alone CNT-sheet/gel-composite electrodes. Body-surface electrocardiographs were clearly detected by both gel probes.

### Admittance measurement

Figure 1g shows the comparison of the admittance among the materials in a wide frequency range. We used electrodes made of CNT/gel, graphite/gel, Au/gel and Al/gel to compare the admittance of the materials in the vertical direction. To prepare CNT/gel electrode, we formed hydrogel on our conductive CNT sheet. Graphite/gel electrode was made of graphite electrode, which was typically used in medical practice(Vitrode V, Nihon Kohden). Commercial-type hydrogel on this graphite electrode was removed by swelling in pure water. Next, we formed hydrogel on bare graphite electrode. Au/gel and Al/gel electrode was prepared on polyimmide substrate. By the same way, hydrogel was formed. We measured the impedance between the electrodes using an LCR meter (E4980, Agilent), to avoid damages of the electrodes by continuous impedance measurements. The conductive gel-composite electrode is used as the interface between the electronic amplifier and an adjacent cell or tissue. In this instance, admittance in the vertical direction is important rather than that in the traverse direction.

In the structure of the CNT/gel electrodes, large capacitance is generated because of the electronic double layer phenomenon. Reactance *X* is determined by the following equation: 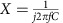, where *C* is the capacitance and *f* is the frequency. A large *C* leads to small reactance (large admittance: admittance is defined as the reciprocal of reactance). Therefore, owing to the large *C*, large admittance can be maintained even in the low-frequency range, as shown in Fig. 1g.

A large capacitance in the system results in a resistance-capacitance (*RC*) time constant, thus leading to a signal propagation delay. On the other hand, the biological signals from a living body are within 1 kHz. Therefore, the propagation delay originating from a large *RC* does not affect the detection of biosignals. The AC admittance of the CNT/gel electrodes can be observed in Supplementary Fig. 3, obtained from four different samples. The magnified image is also shown in the inset.

## Additional information

**How to cite this article:** Sekitani, T. *et al.* Ultraflexible organic amplifier with biocompatible gel electrodes. *Nat. Commun.* 7:11425 doi: 10.1038/ncomms11425 (2016).

## Supplementary Material

Supplementary Information Supplementary Figures 1-23, Supplementary Tables 1-3.

## Figures and Tables

**Table 1:** Average irritant ranking score, comparison with negative control and degree of irritant for six samples.

**Sample (four-week implantation)**	**Average irritant ranking score**	**Δ Between test sample and control**	**Non-irritant (0.0–2.9)Slight irritant (3.0–8.9)Moderate irritant (9.0–15.0)Severe irritant (>15)**
Negative control	4.0	—	—
Gel	18.0	14.0	Moderate irritant
Gel+IL	15.5	11.5	Moderate irritant
Gel+CNT	18.3	14.3	Moderate irritant
Gel+IL+CNT	15.3	11.3	Moderate irritant
Au	22.0	18.0	Severe irritant

CNT, carbon nanotube; gel, polyrotaxane-based gel with movable cross-linker (cyclodextrin/polyethylene); IL, ionic liquid; Negative control, high-density polyethylene sheet.

The assays were quantified based on ISO 10993-6:2007, Biological Evaluation of Medical Devices—Part 6: Tests for Local Effects After Implantation.
