# Supplementary material for: Ultraflexible organic amplifier with biocompatible gel electrodes
Source: Nat Commun. 2016 Apr 29;7:11425. doi: 10.1038/ncomms11425 (PMC5411732; doi:10.1038/ncomms11425)
Supplement: Supplementary Information — Supplementary Figures 1-23, Supplementary Tables 1-3. [file ncomms11425-s1.pdf]

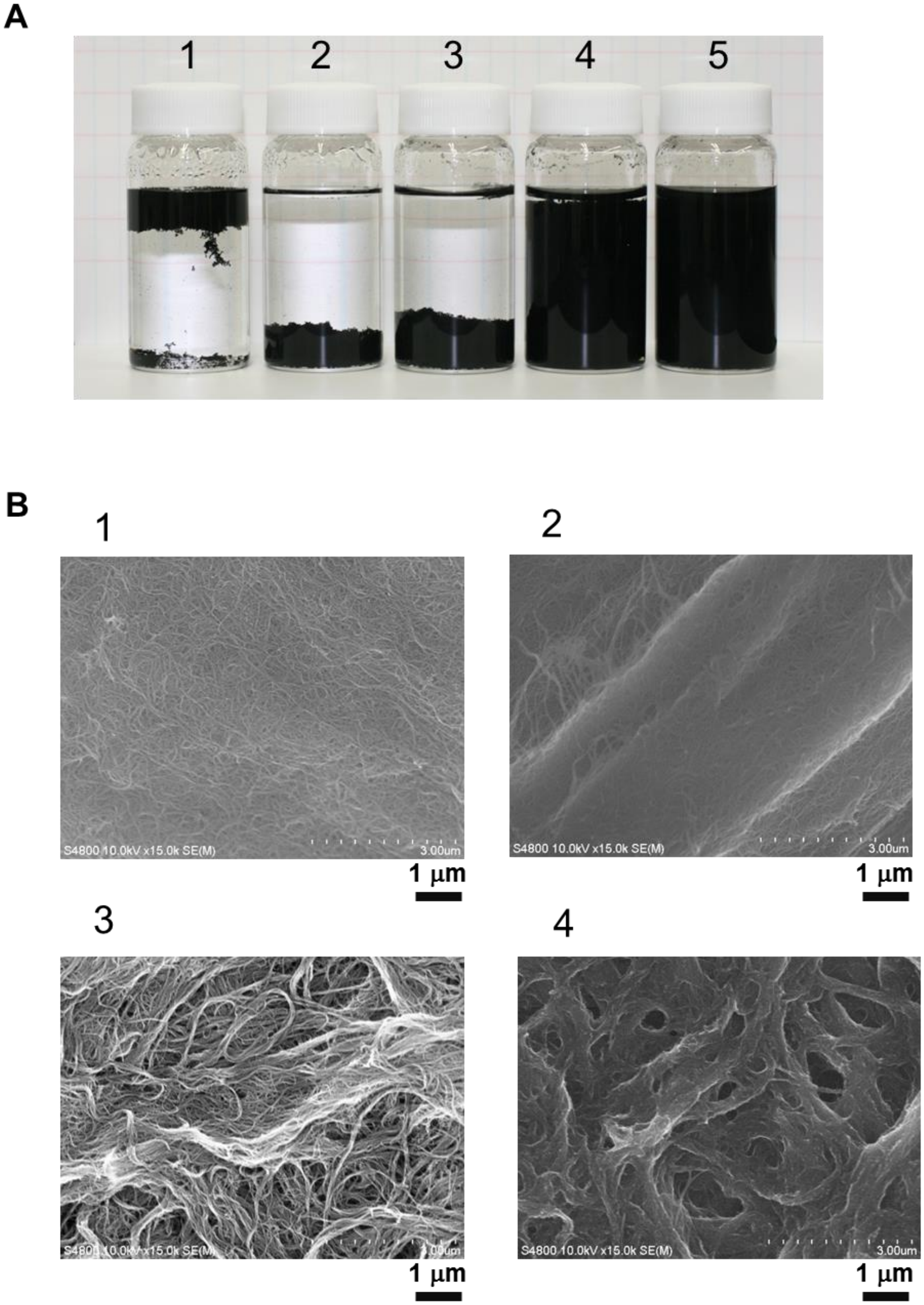

Supplementary Figure 1

Water dispersibility of carbon nanotubes (CNTs)

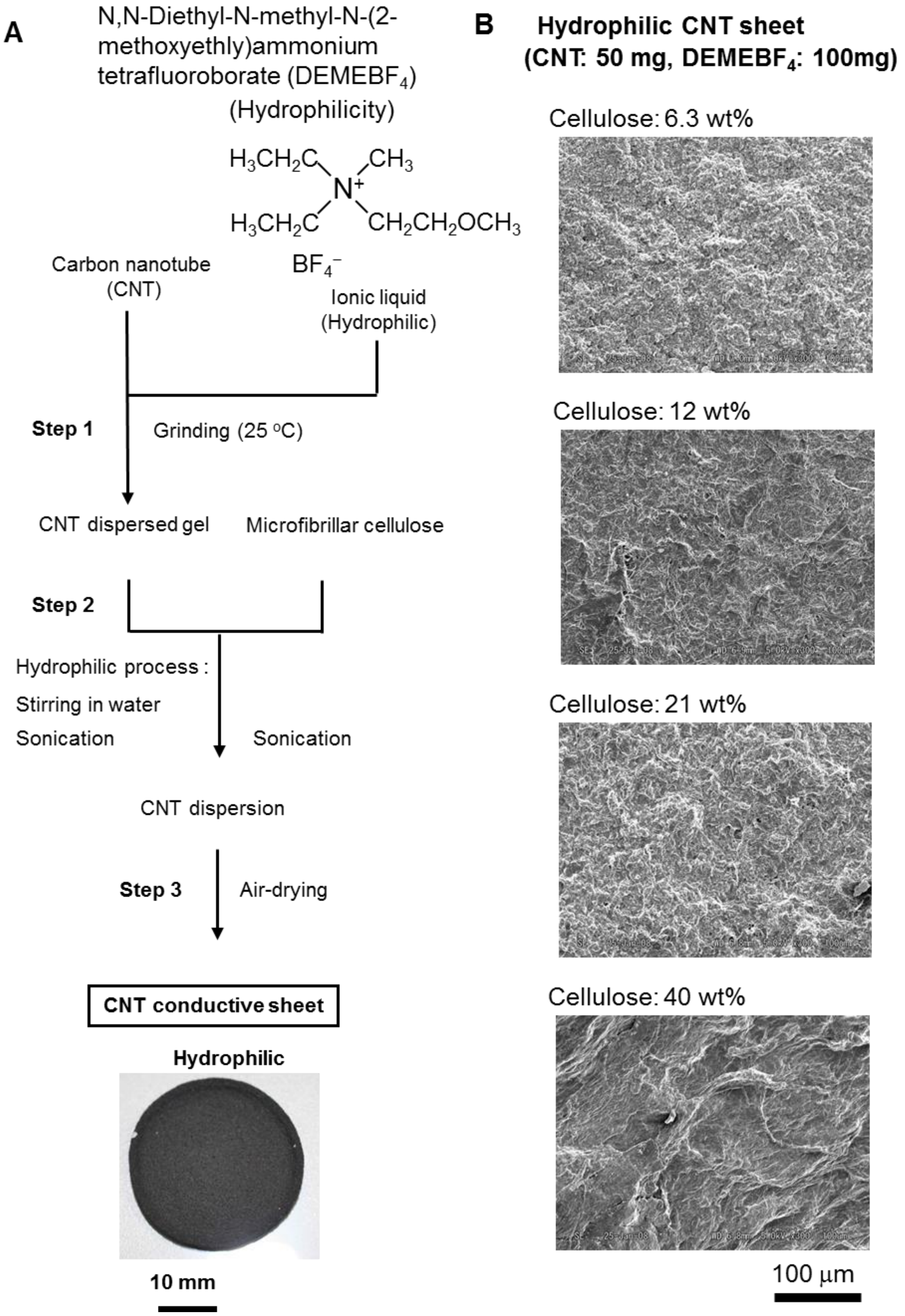

Supplementary Figure 2

Manufacturing process of CNT composite sheet (CNT sheet)

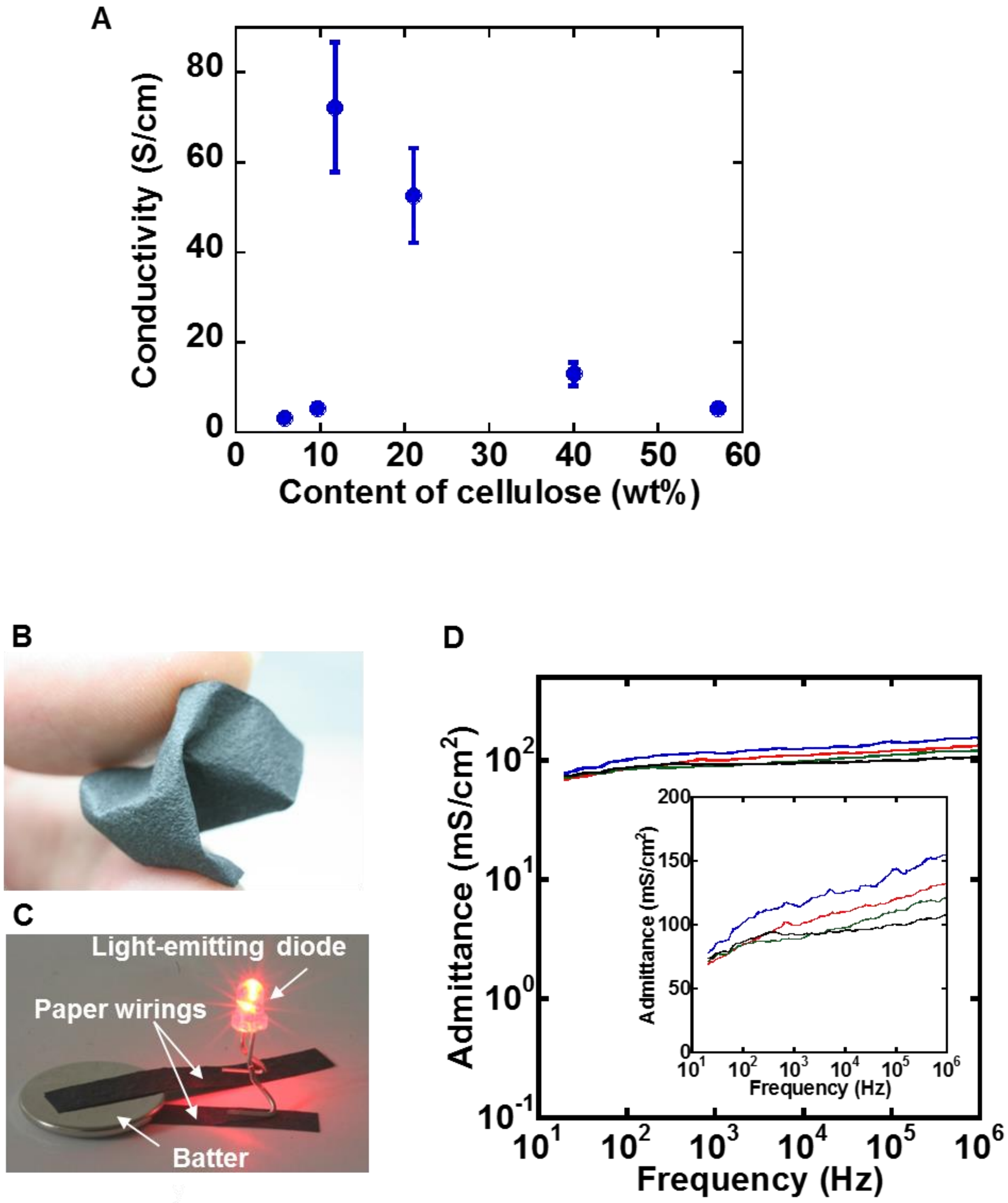

Supplementary Figure 3

Characteristics of CNT composite sheet (CNT sheet)

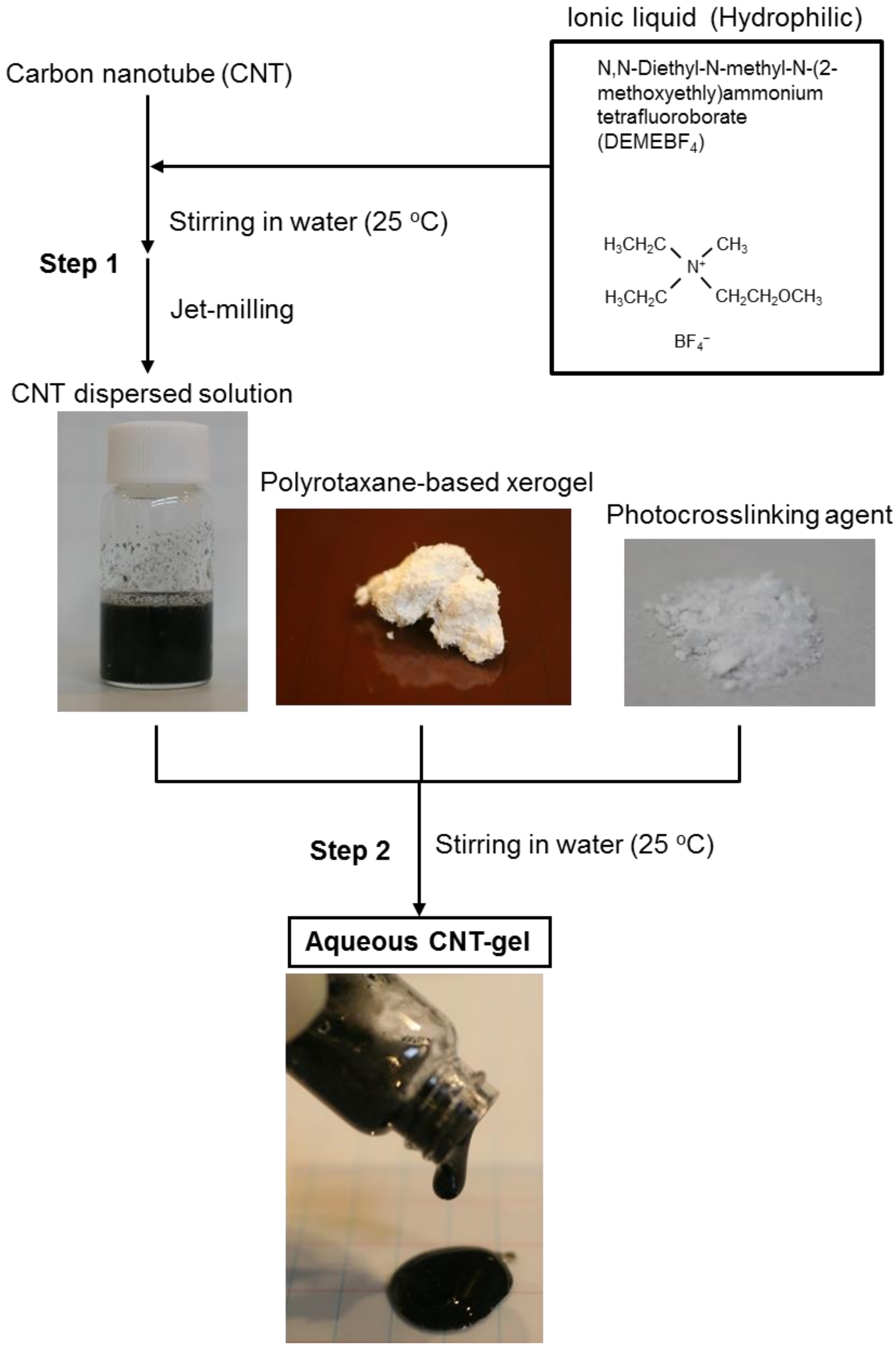

Supplementary Figure 4

Another manufacturing process of CNT/gel composite 1

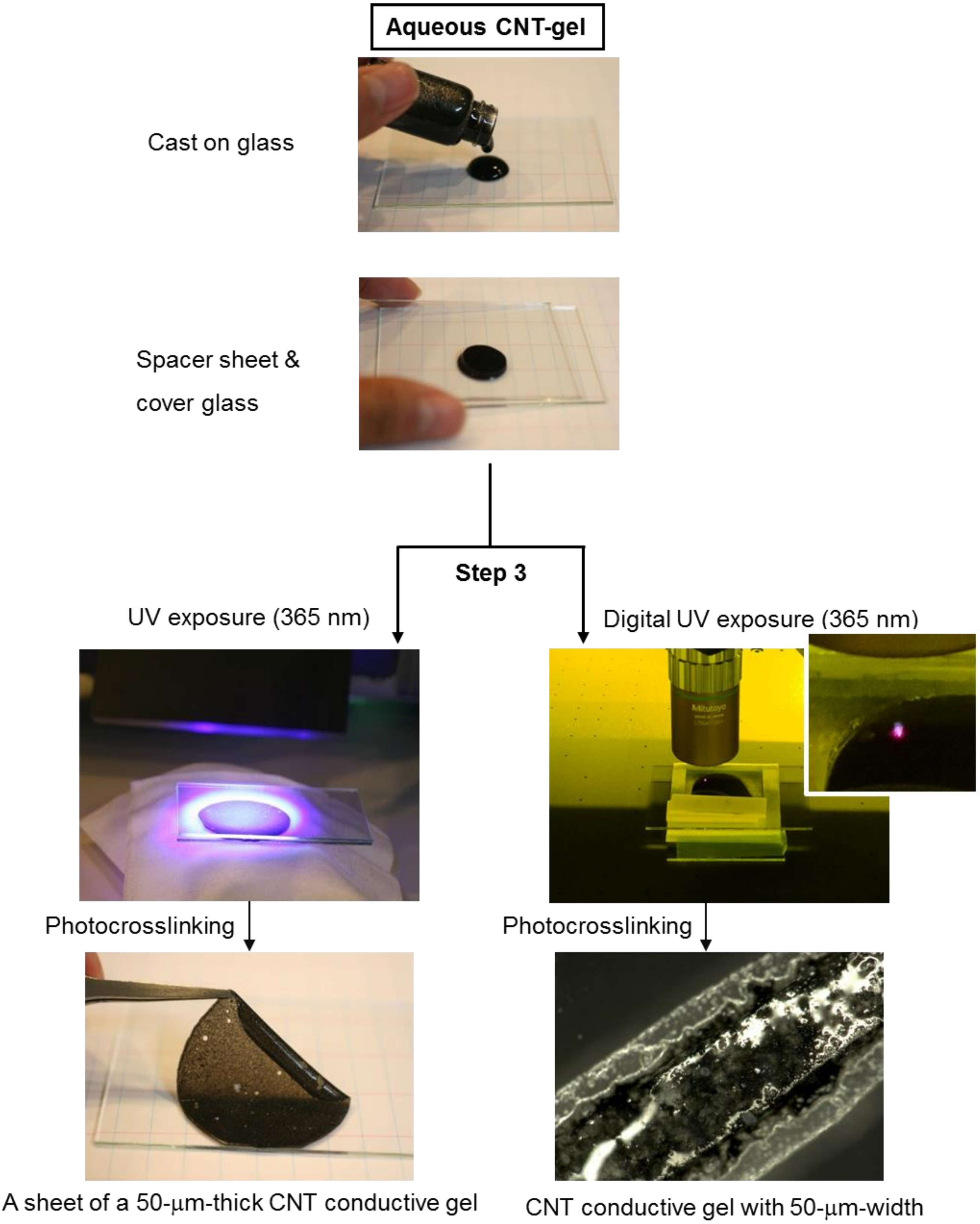

Supplementary Figure 5

Another manufacturing process of CNT/gel composite 2

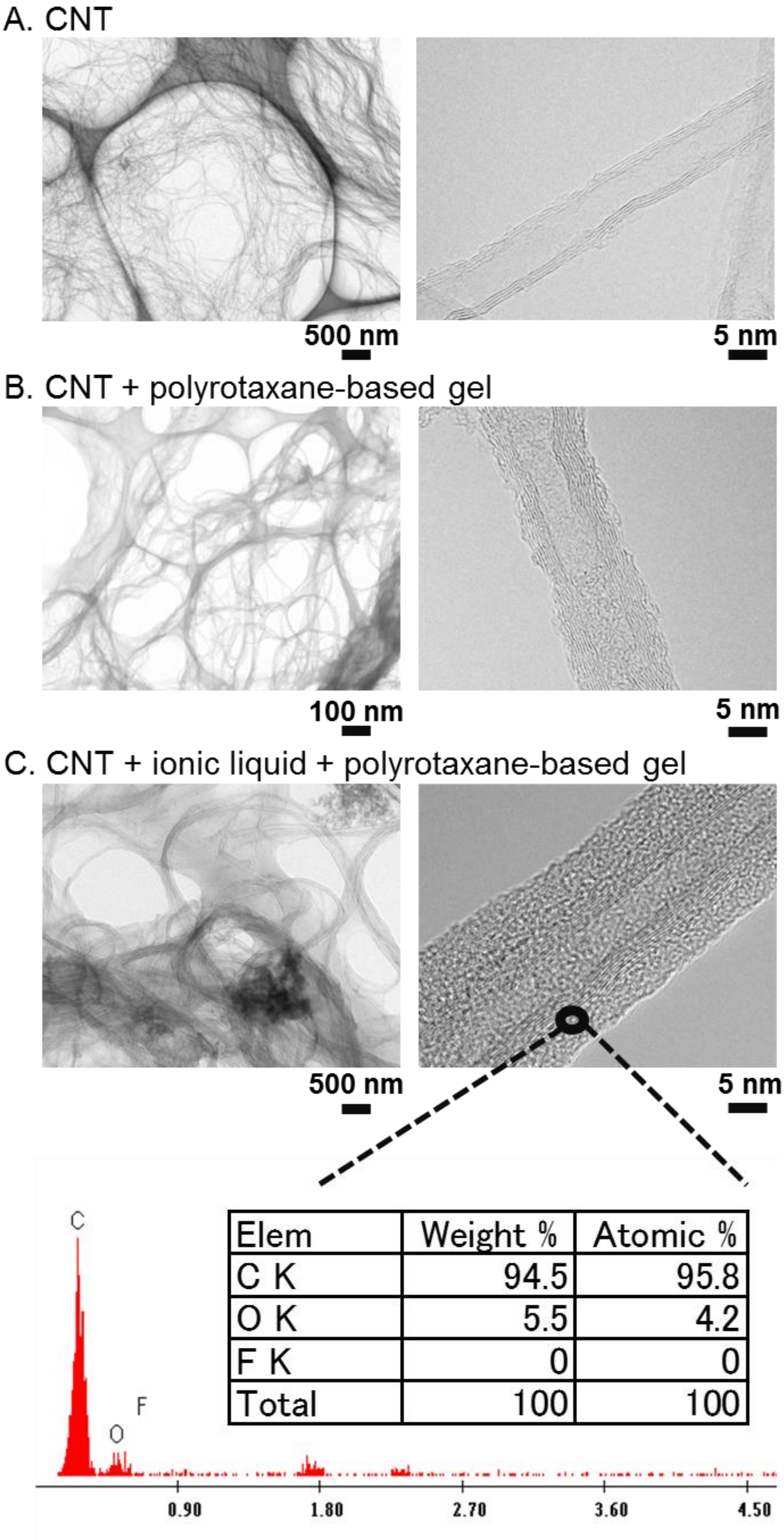

Elem	Weight %	Atomic %
C K	94.5	95.8
O K	5.5	4.2
F K	0	0
Total	100	100

Supplementary Figure 6

Transmission electron microscopy (TEM) observation of CNT/rotaxane composite

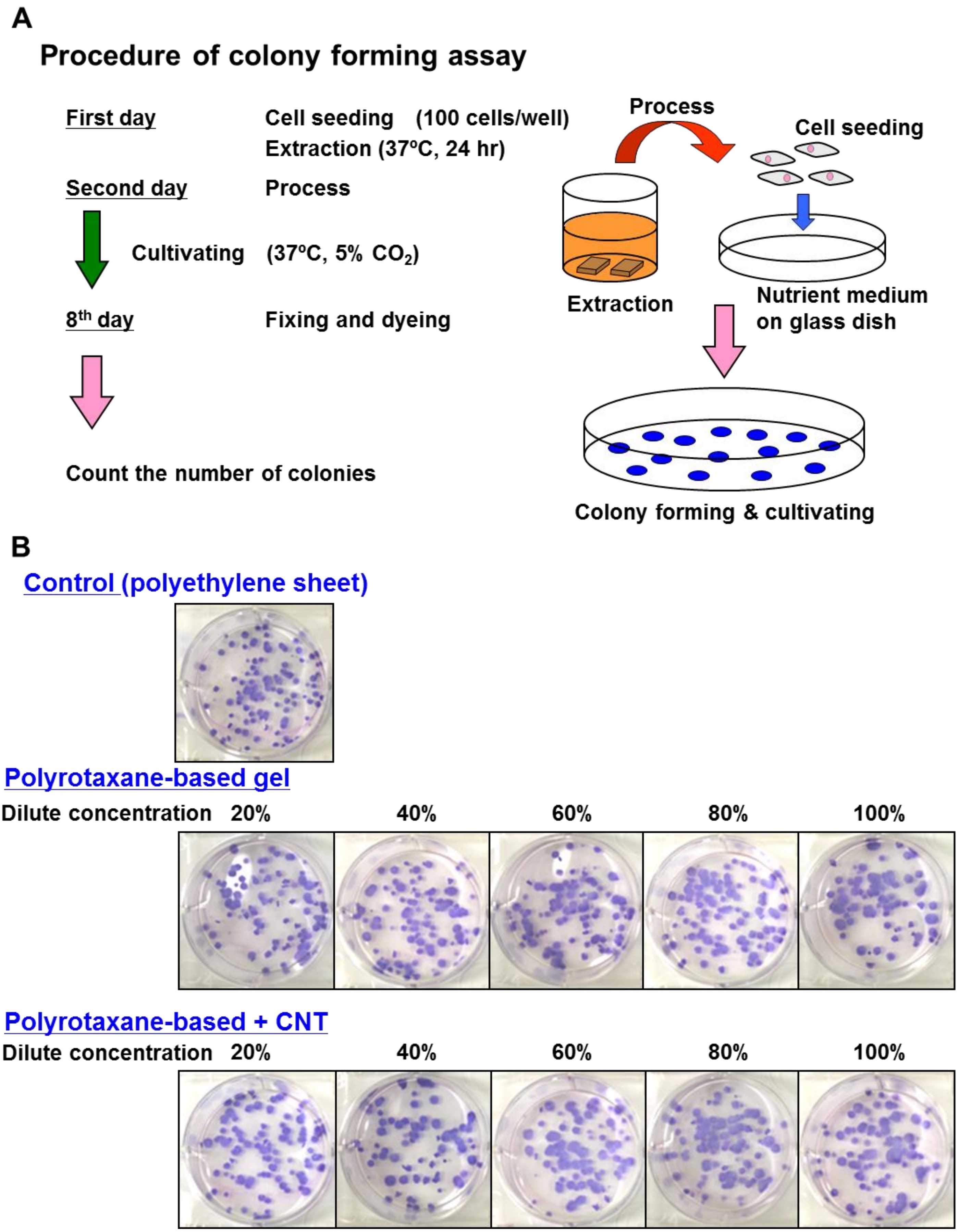

Supplementary Figure 7

Colony-forming assay for cytotoxic evaluation

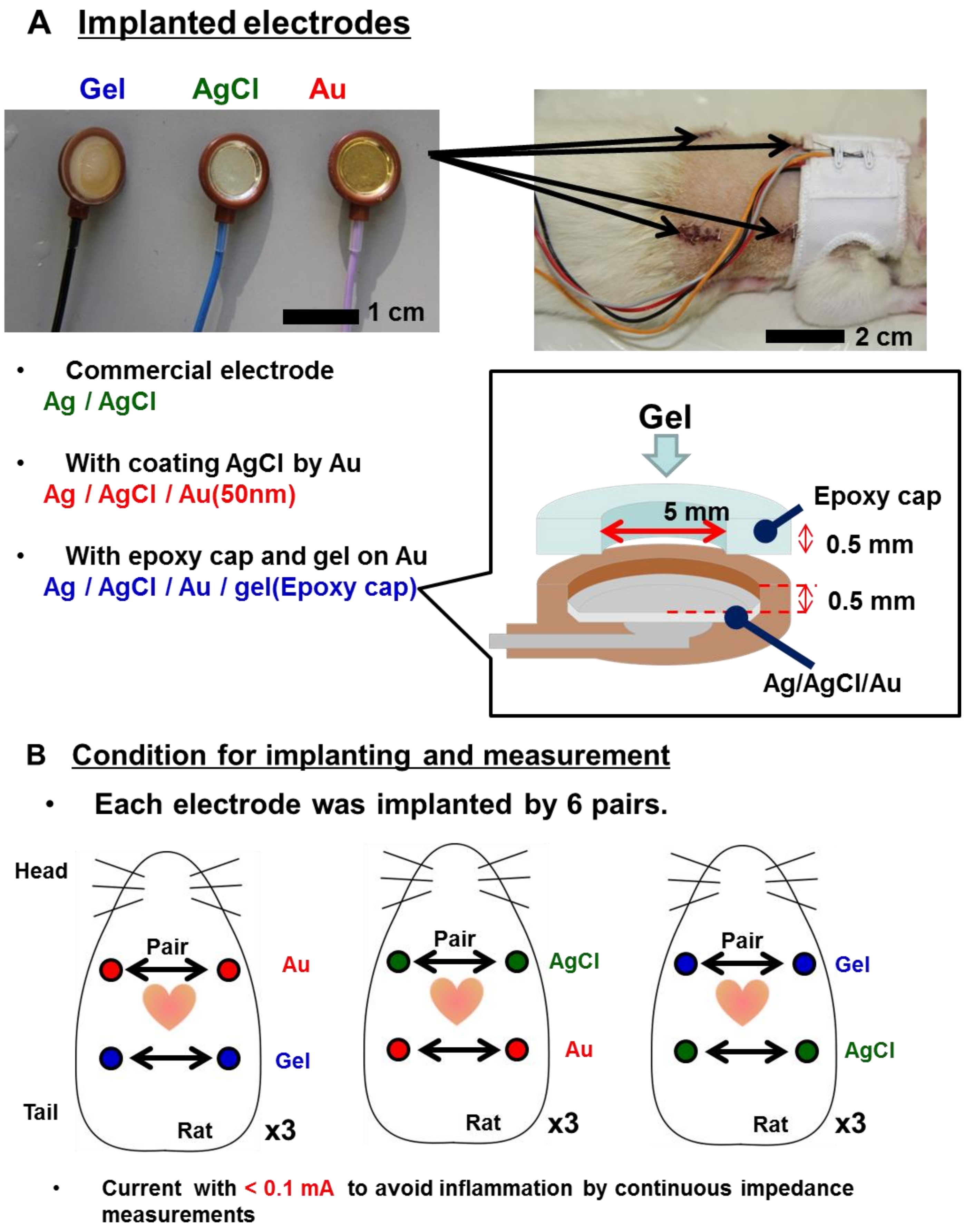

Supplementary Figure 8

Implant assay for biocompatibility evaluation

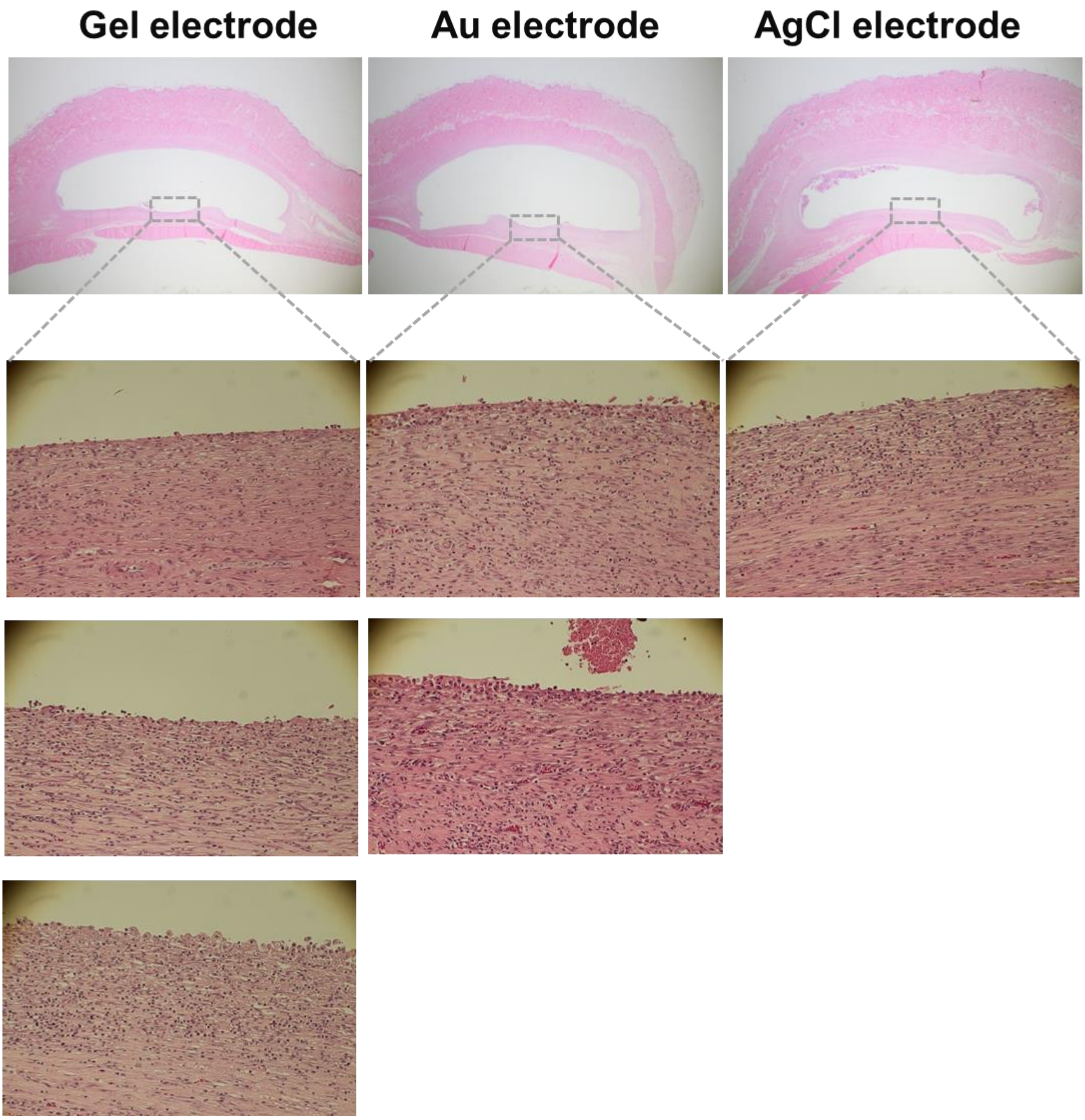

Supplementary Figure 9

Magnified cross-sectional images of the graft pathology by staining a subcutaneous tissue after an electrode was explanted

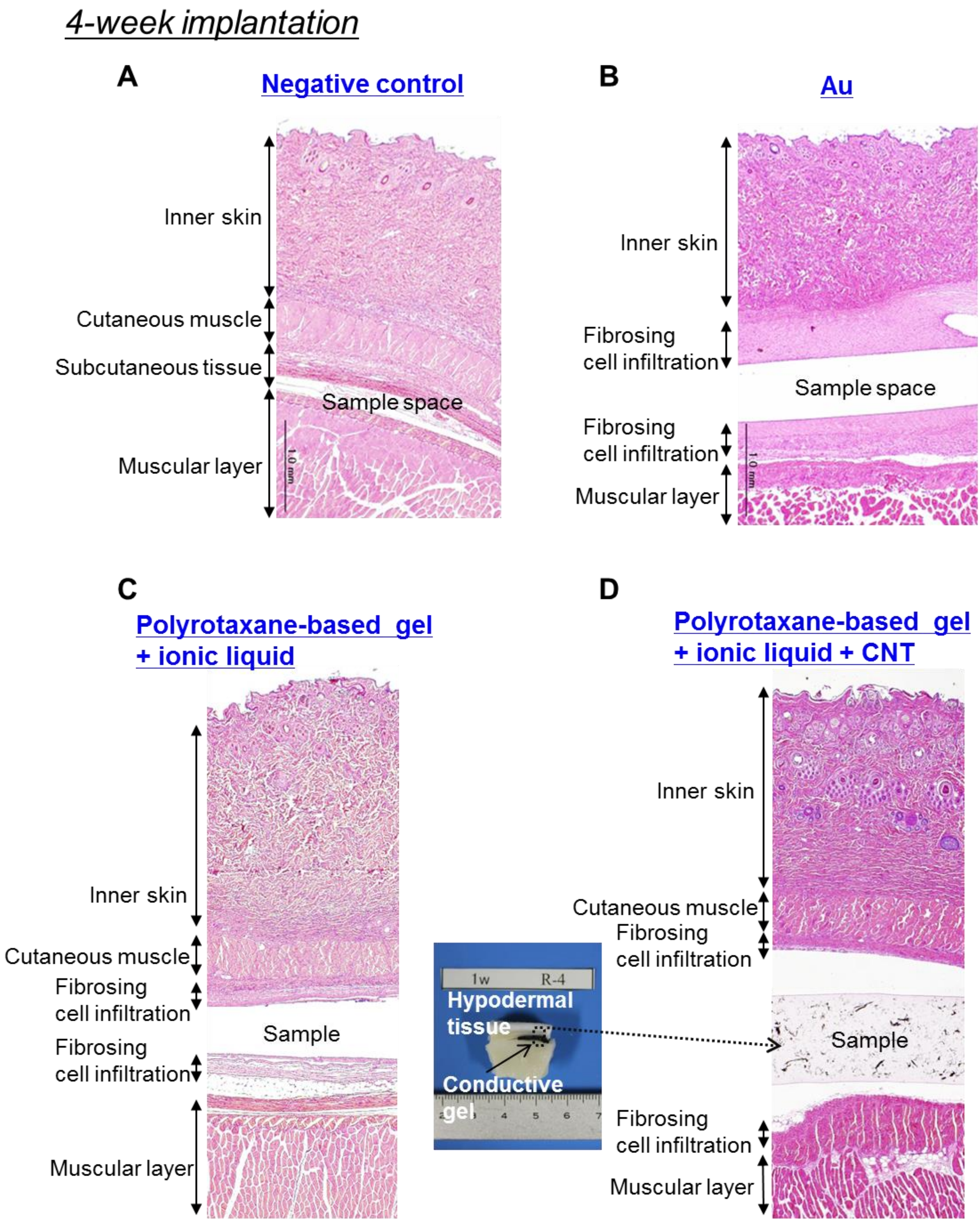

Supplementary Figure 10

Graft pathologies of the four different samples after implantation for four weeks

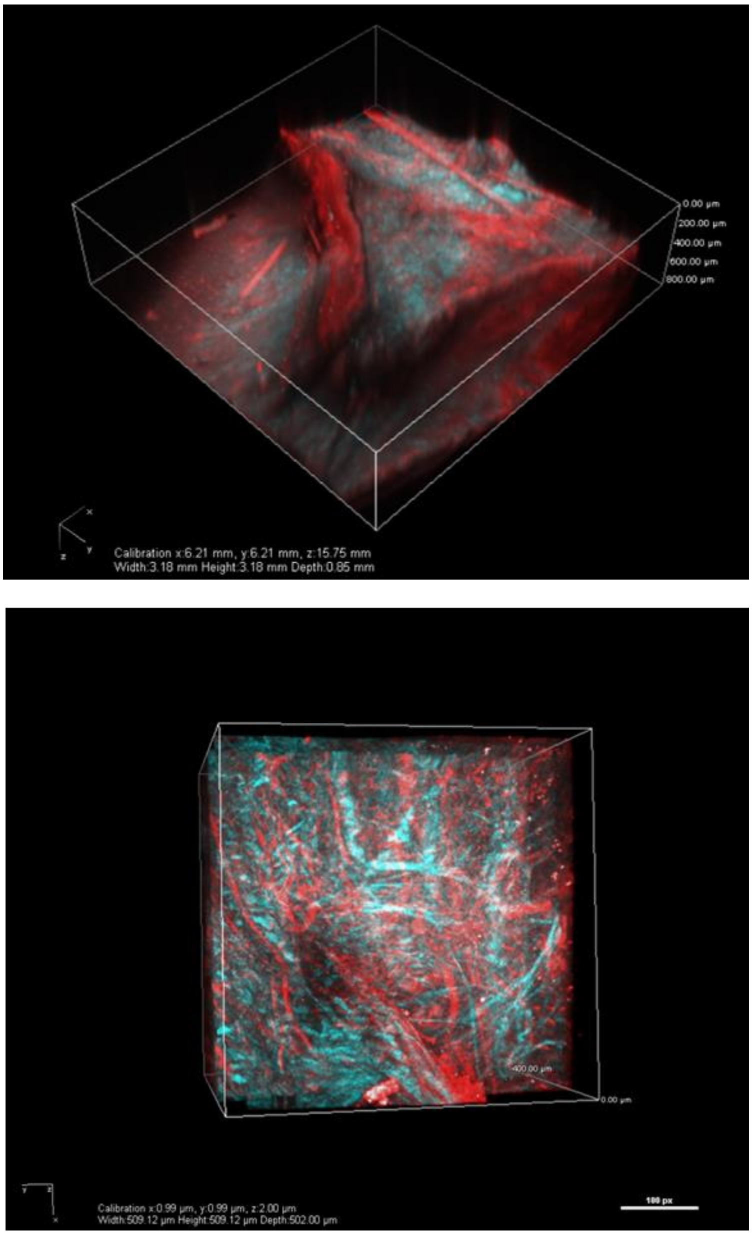

Supplementary Figure 11

Procedures of perfusion fixation, tissue-cleaning method, and evaluation using multiphoton microscopy

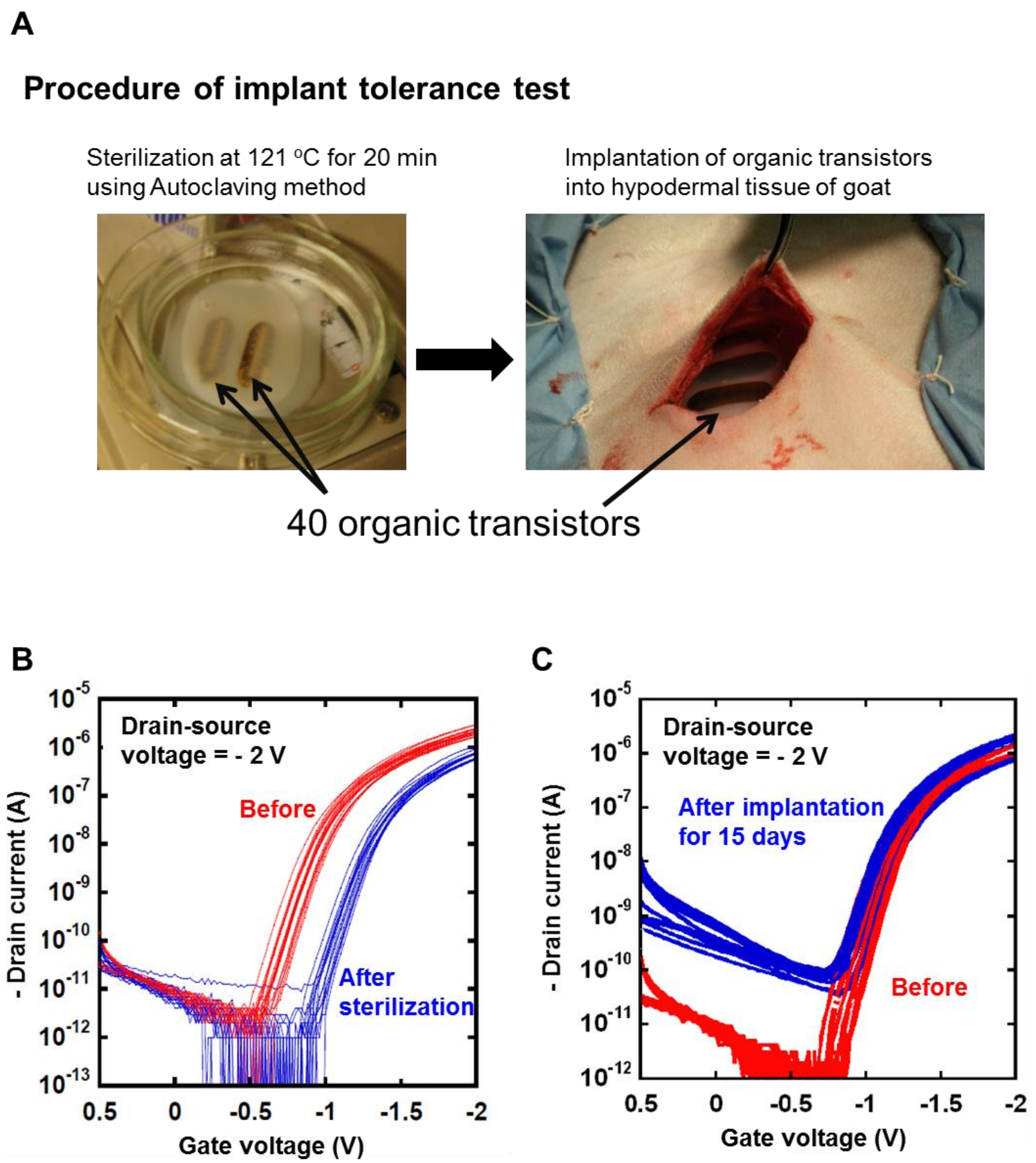

Supplementary Figure 12

In vivo implant tolerance of organic transistors

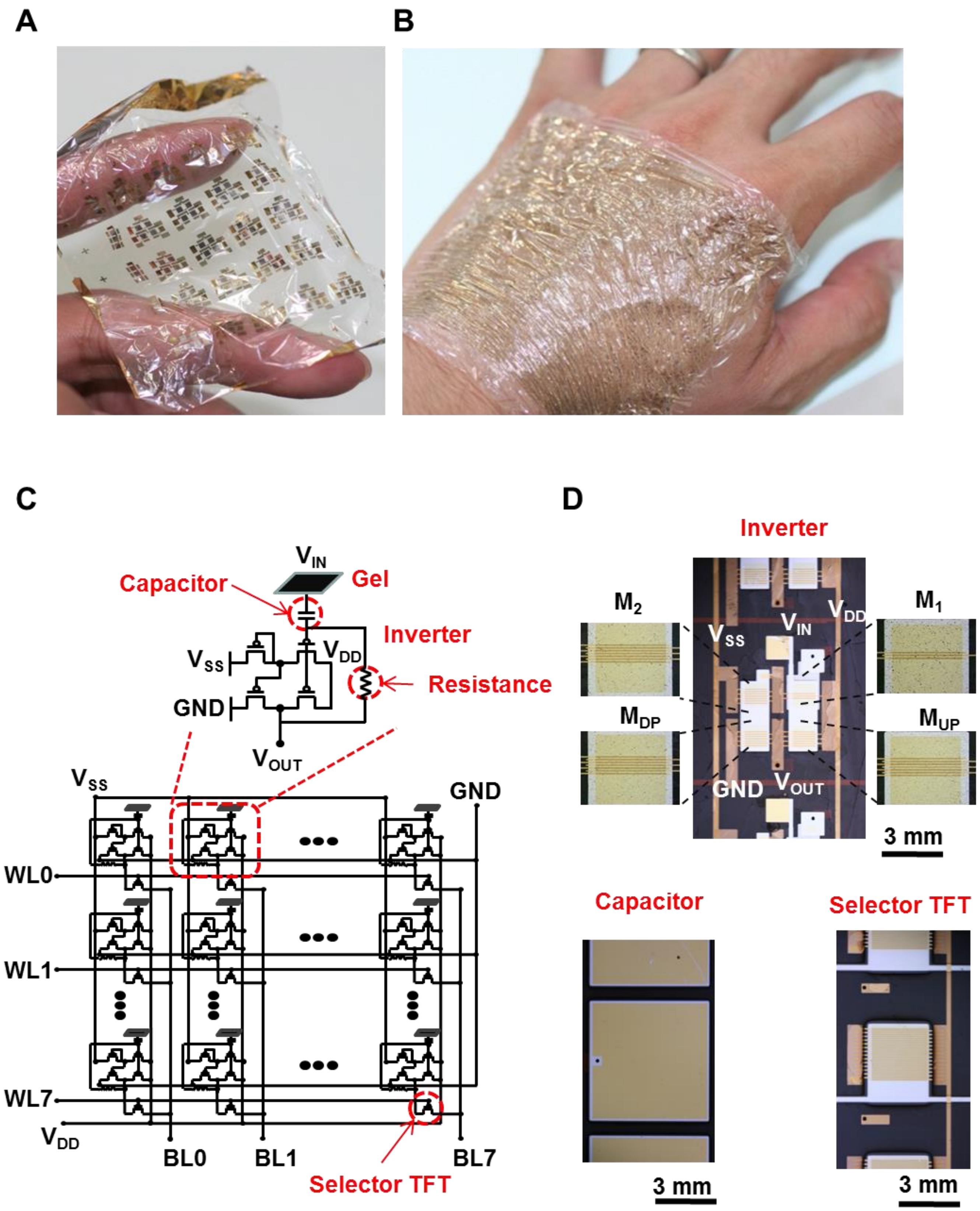

Supplementary Figure 13

1.2-μm-thick organic circuits

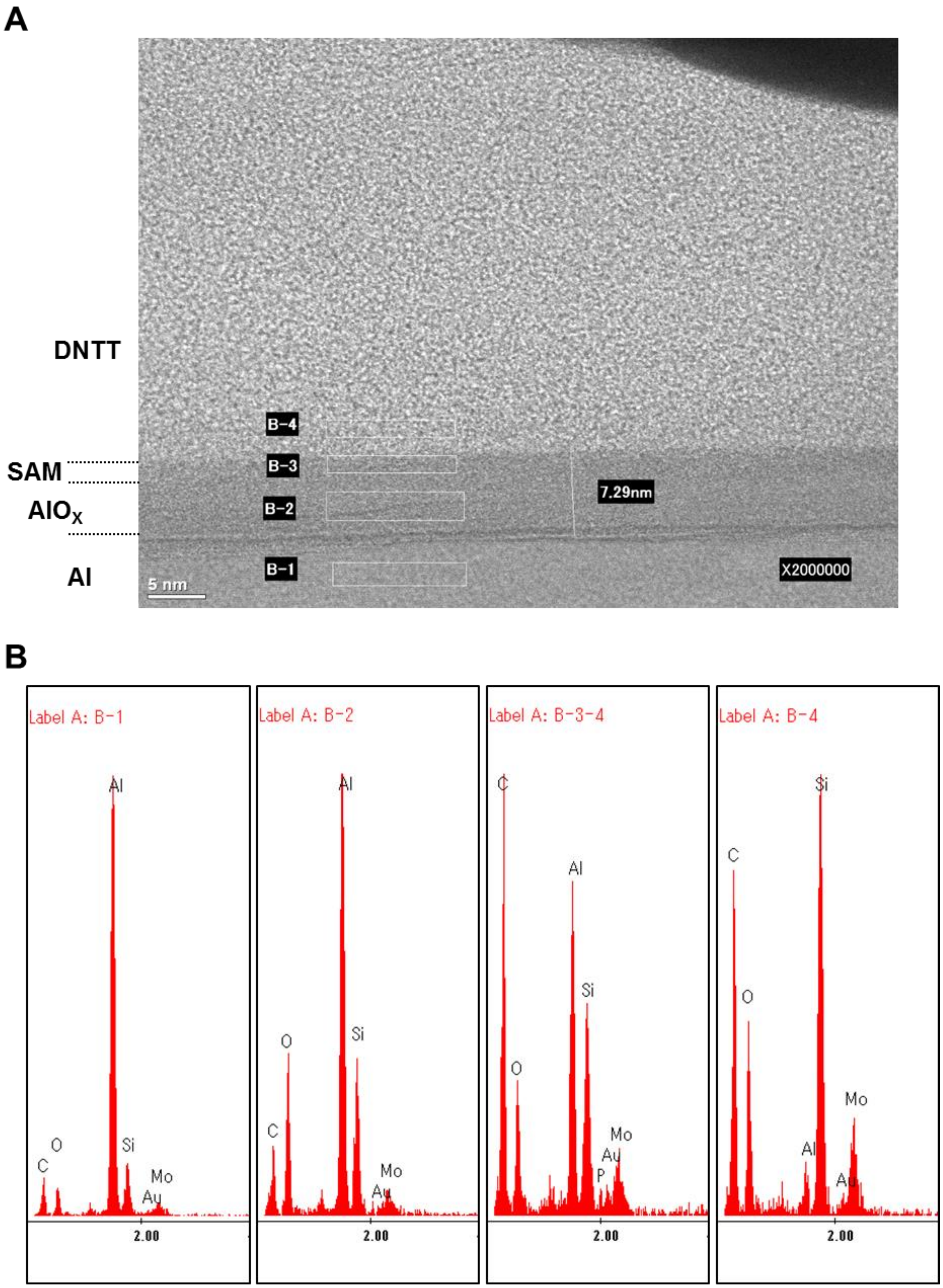

Supplementary Figure 14

Material profiling and cross-sectional image of organic transistors on a 1.2-μm-thick substrate using ultrahigh resolution scanning TEM (STEM) and EDX analysis system

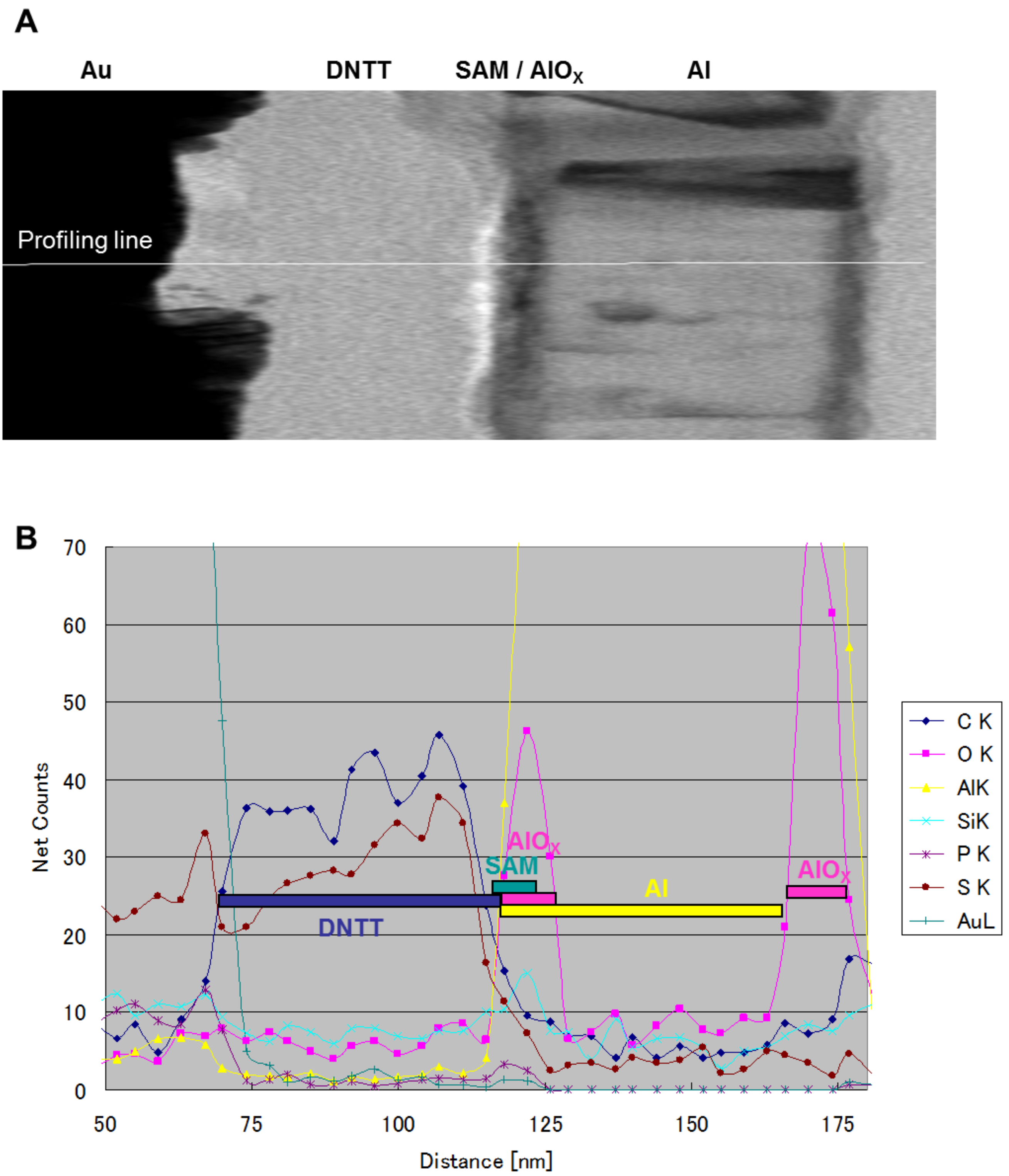

Supplementary Figure 15

Material profiling and cross-sectional image of organic transistors on a 1.2-μm-thick substrate using ultrahigh resolution scanning TEM (STEM) and EDX analysis system

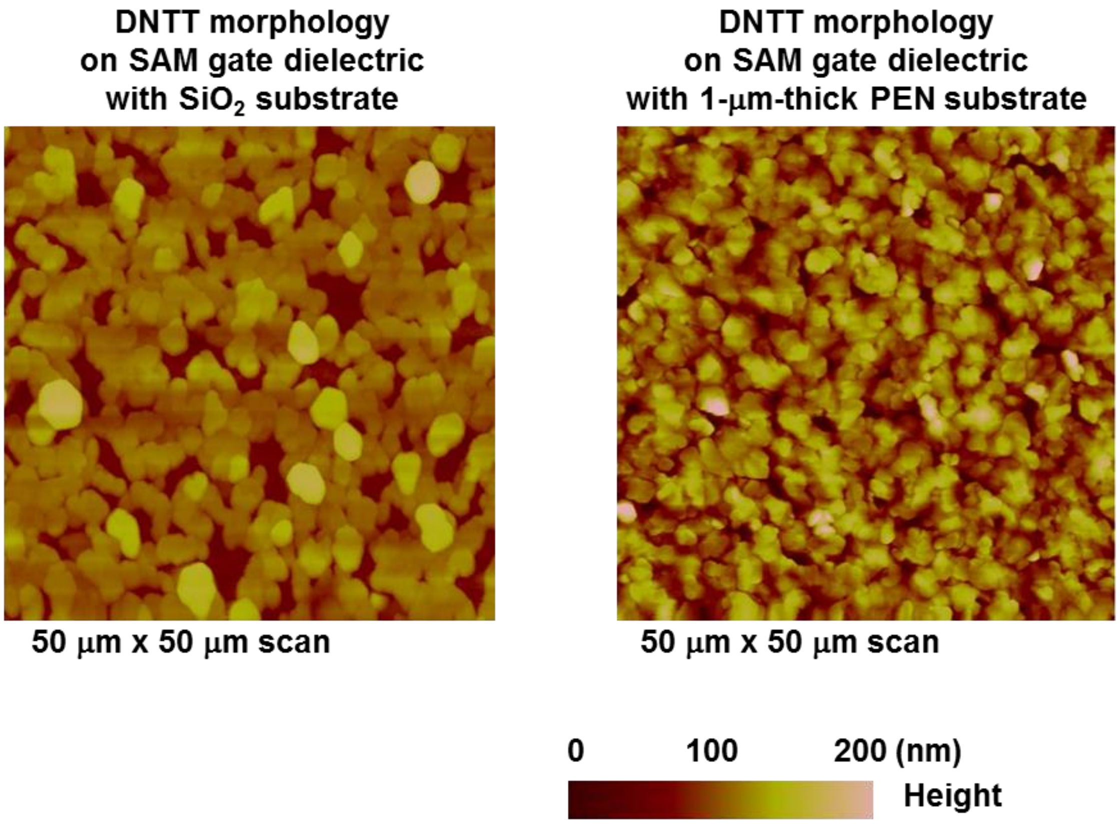

Supplementary Figure 16

Morphology of DNTT on SAM

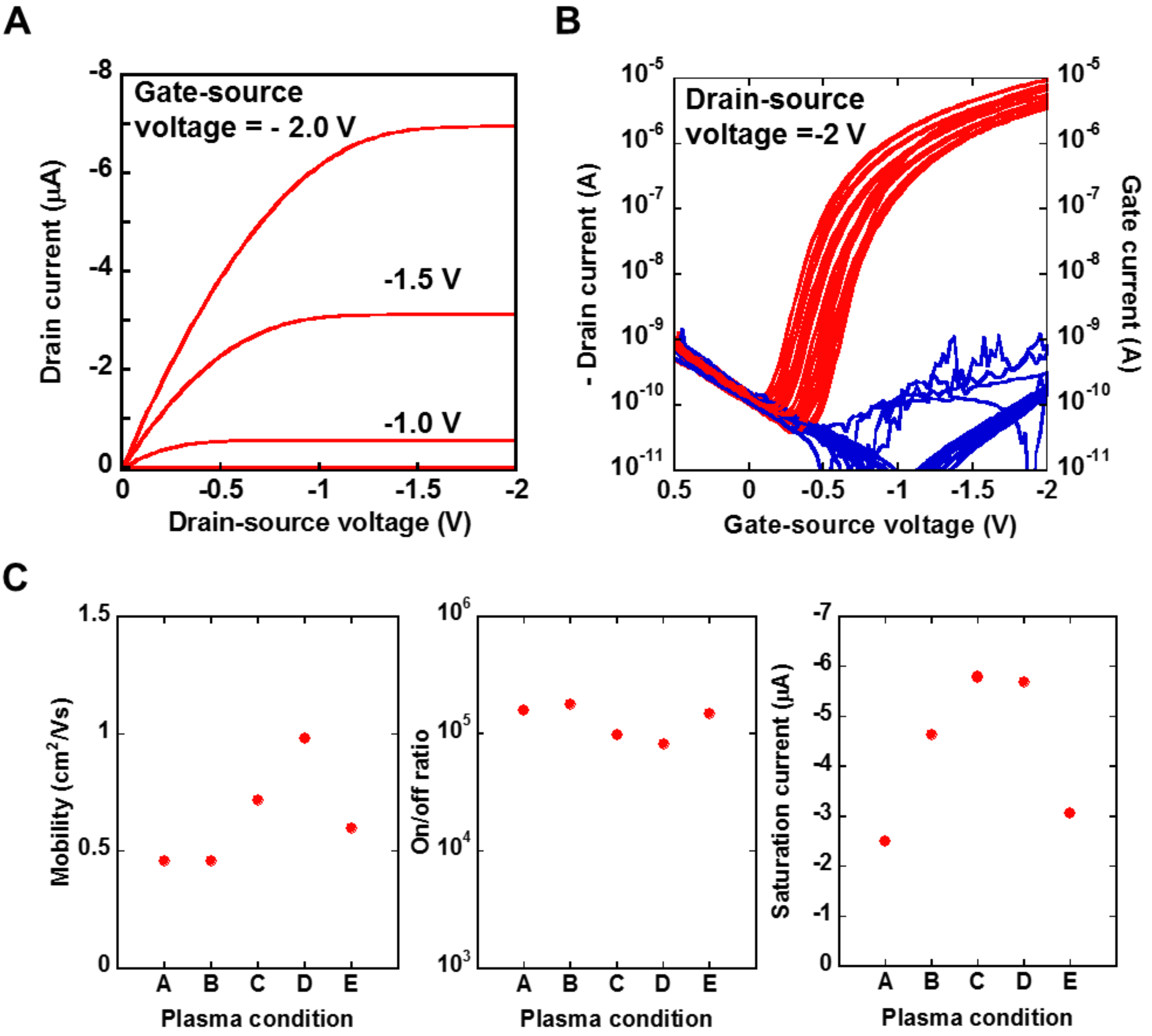

Supplementary Figure 17

Optimization of the manufacturing process on a 1-μm-thick substrate

A

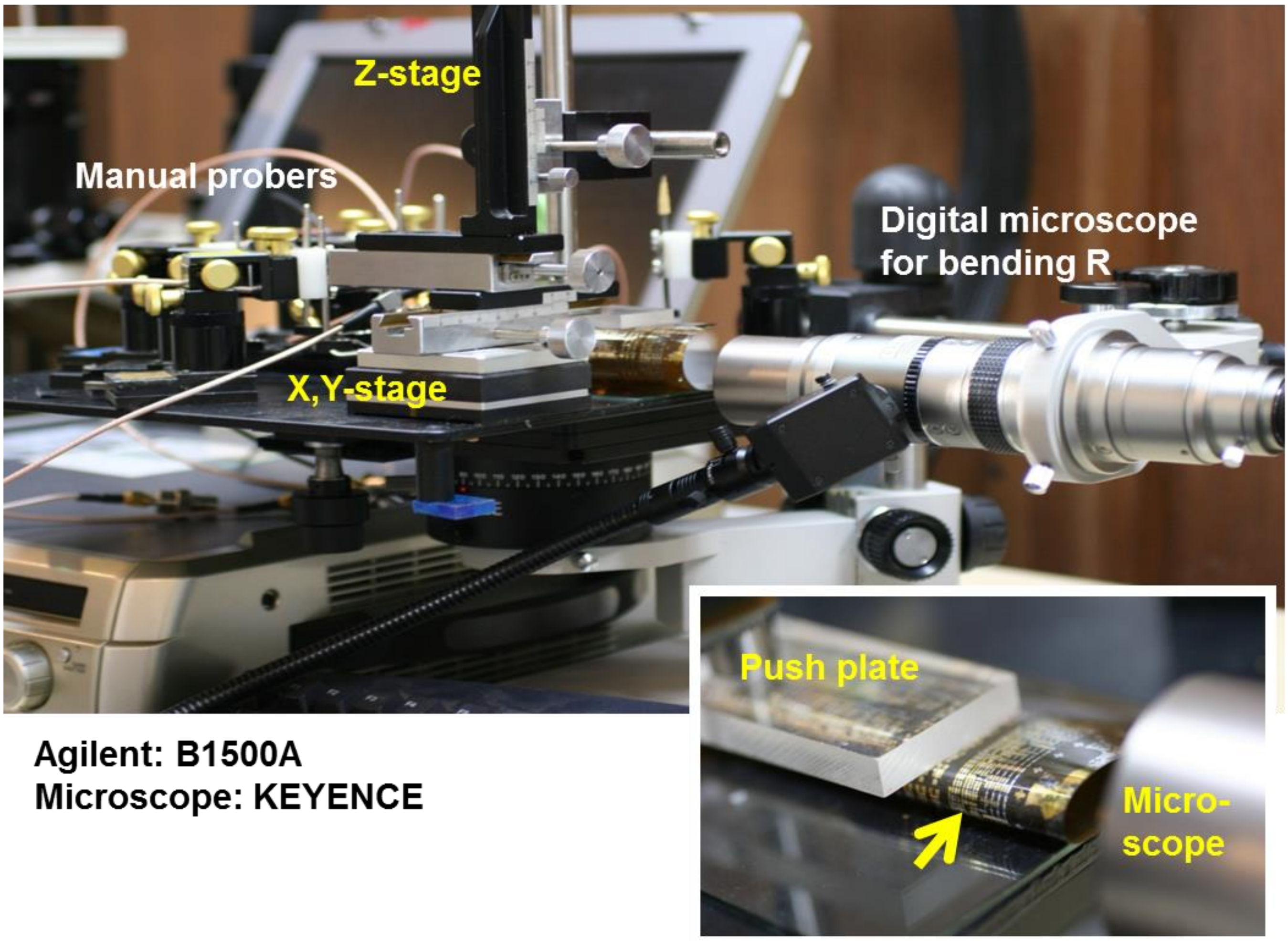

Supplementary Figure 18

Bending test

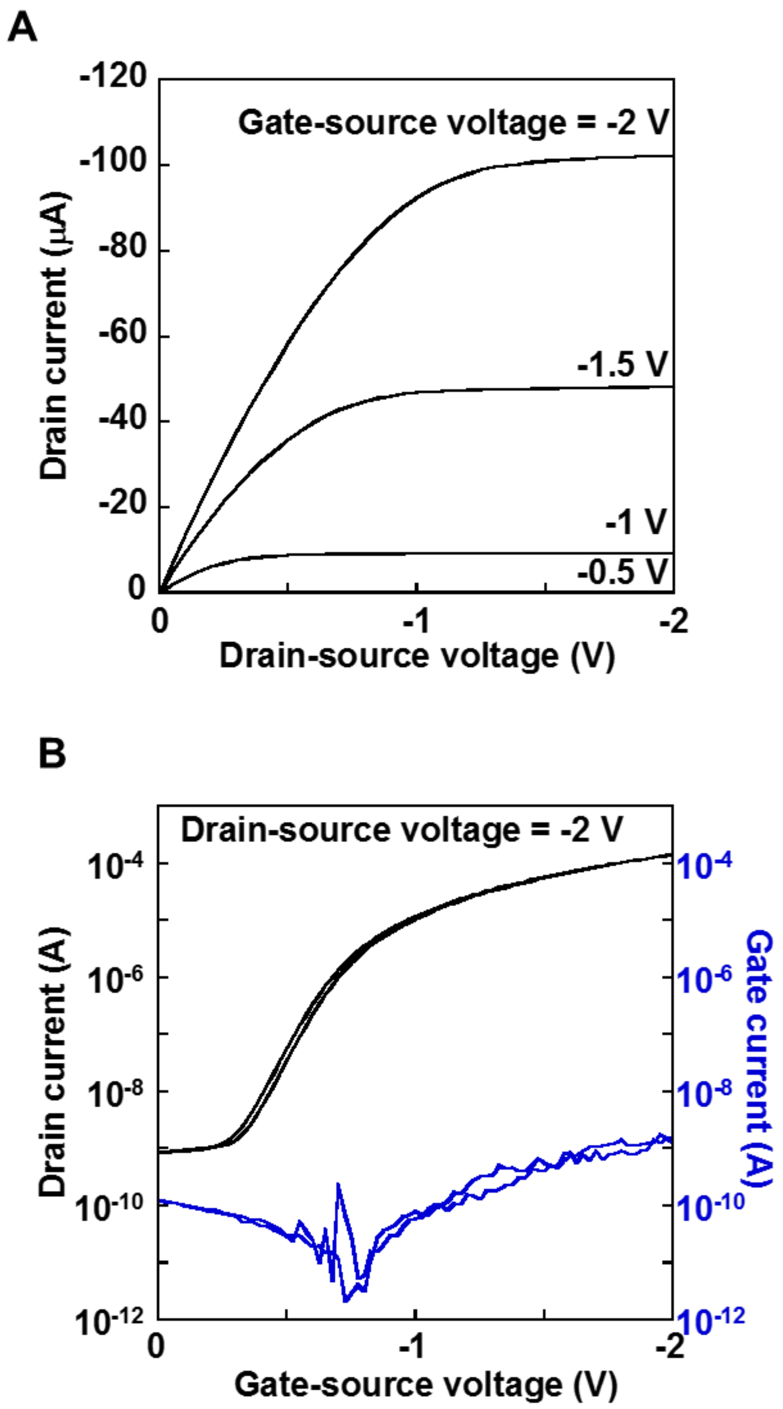

Supplementary Figure 19

Design of organic transistor for higher amplifier gain

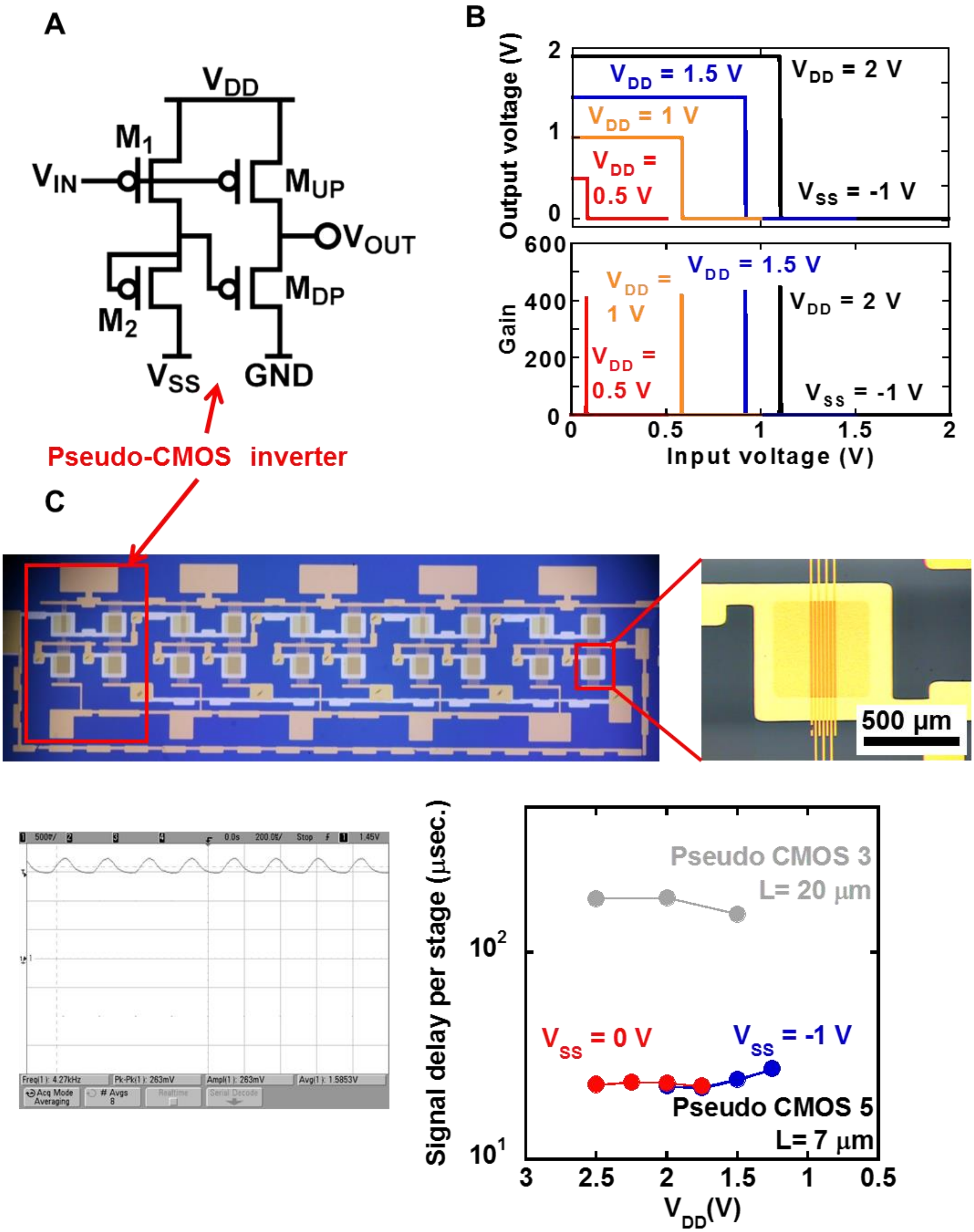

Supplementary Figure 20

Pseudo-CMOS inverter

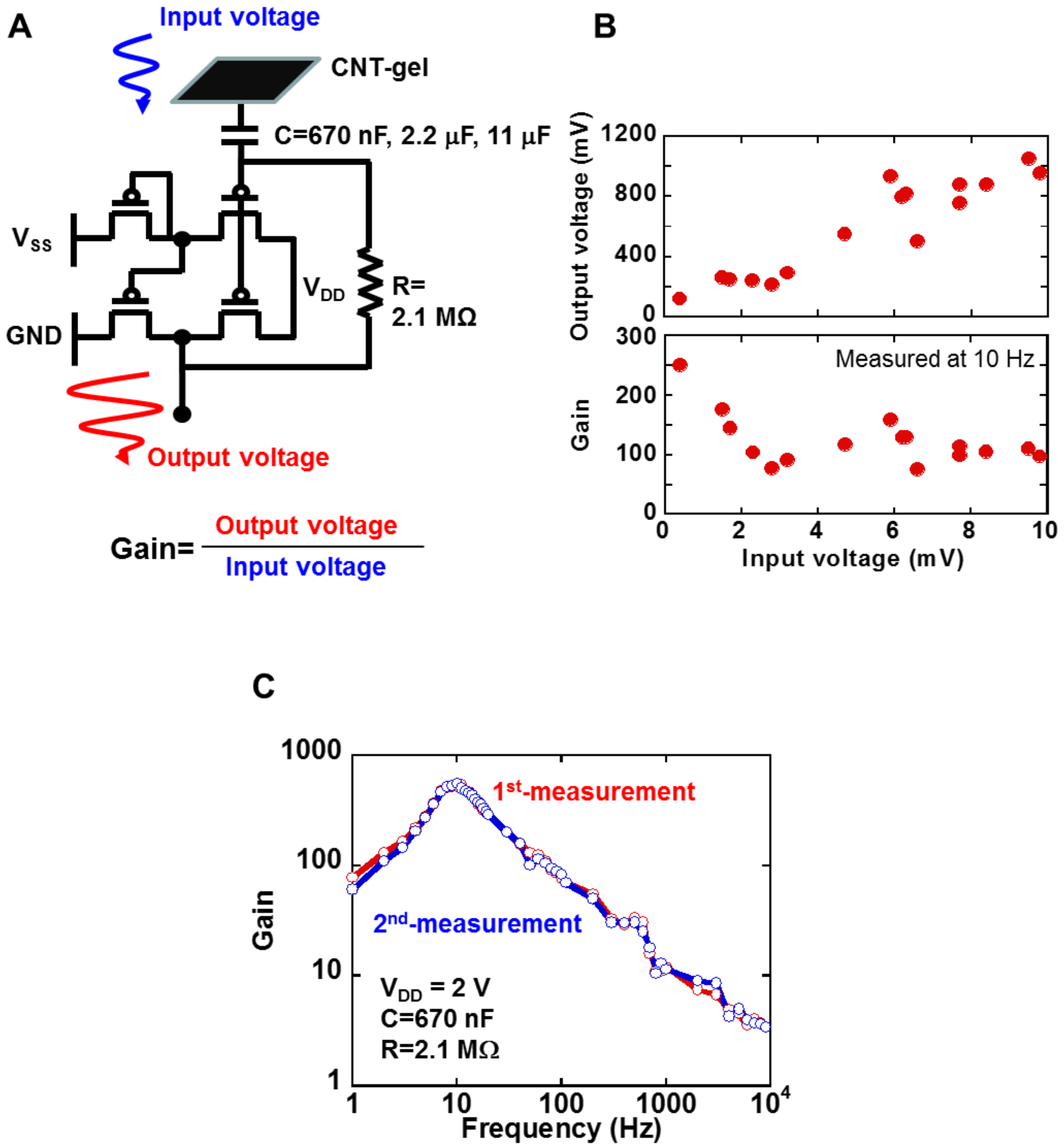

Supplementary Figure 21

Signal gain and frequency response of organic amplifiers

Ischemia-induced myocardial infarction

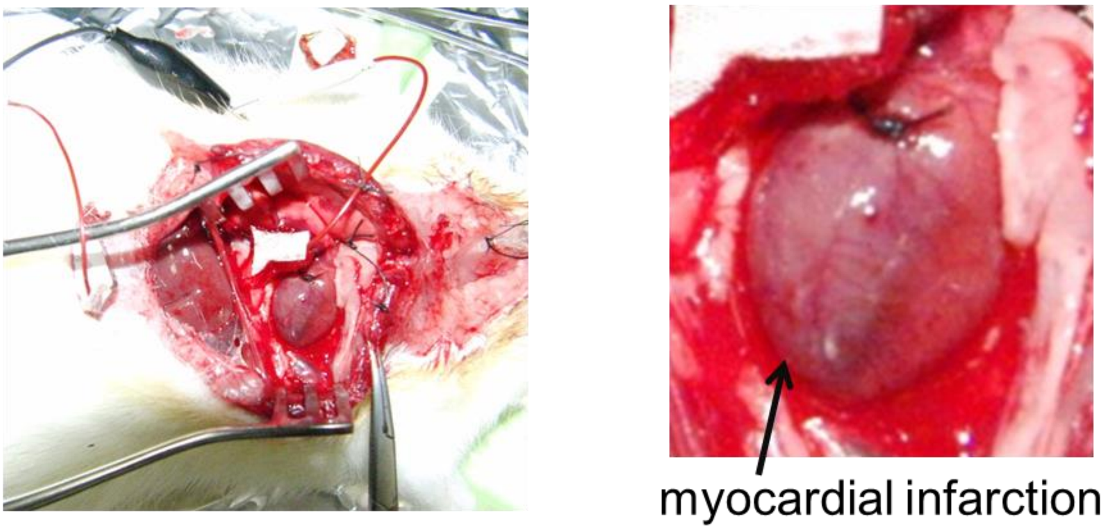

Supplementary Figure 22

Detection of ischemic state due to myocardial infarction

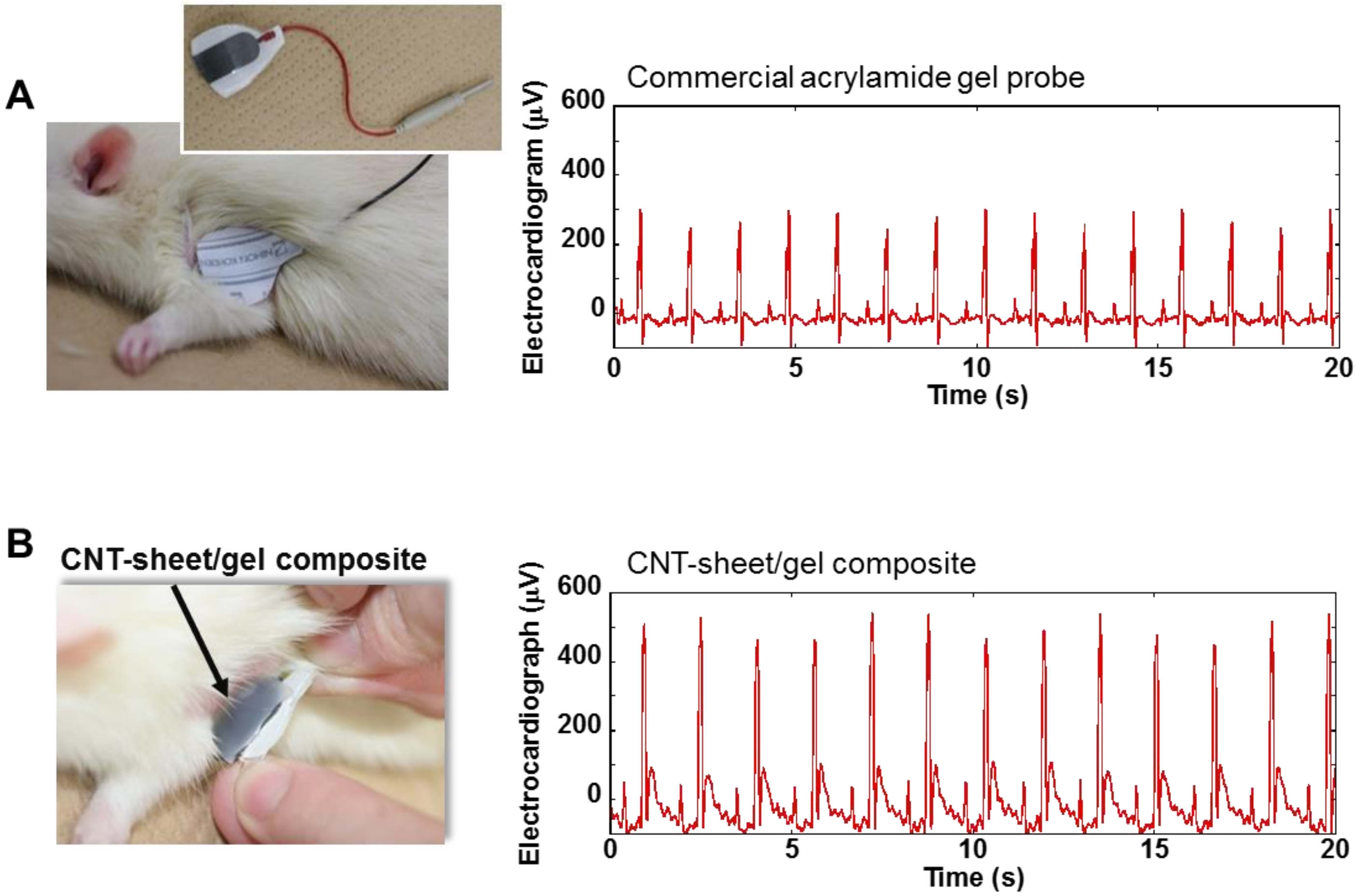

Supplementary Figure 23

Effects of the CNT-sheet/gel-composite electrode

A

(I) Colony forming ability (%) :

$$\frac{\text{The number of colonies w/o processed well}}{\text{The number of cell seeding}} \times 100$$

(II) Rate of relative colony formation (%) :

$$\frac{\text{The number of colonies with processed well}}{\text{The number of colonies with control well}} \times 100$$

(III)

IC_{50} value	Cell toxicity
Negligible small change	None
Higher than values from positive control B	Slight
Values between positive control A and B	Moderate
Lower than values from positive control A	Severe

B

Rate of relative colony formation and IC_{50} value of V79 cells processed with the following extraction liquids

Sample	Concentration (%)	The number of colonies (count / well)					Rate of relative colony formation (%)	IC_{50} value (%)
		Well 1	Well 2	Well 3	Mean	S.D.		
Negative control (Fresh MO5 culture media)	0	101	90	89	93	7	100.0	–
Gel	20	92	97	92	94	3	101.1	–[a]
	40	97	92	82	90	8	96.8	
	60	97	87	80	88	9	94.6	
	80	94	104	91	96	7	103.2	
	100	75	86	89	83	7	89.2	
Gel + ionic liquid	20	98	101	90	96	6	103.2	
	40	73	91	91	85	10	91.4	
	60	82	97	98	92	9	98.9	
	80	86	80	89	85	5	91.4	
	100	83	80	82	82	2	88.2	
Gel + ionic liquid + CNT	20	99	88	74	87	13	93.5	
	40	83	86	78	82	4	88.2	
	60	72	92	91	85	11	91.4	
	80	95	84	89	89	6	95.7	
	100	97	77	76	83	12	89.2	

a : No decreases were observed on the rate of relative colony formation, as a result that IC_{50} value cannot be estimated.

Rate of relative colony formation and IC_{50} value of V79 cells processed with the following control extraction liquids

Control sample	Concentration (%)	The number of colonies (count / well)					Rate of relative colony formation (%)	IC_{50} value (%)
		Well 1	Well 2	Well 3	Mean	S.D.		
Negative control (Fresh MO5 cultivate media)	0	101	90	89	93	7	100.0	–
Negative control (Polyethylene film)	25	99	83	95	92	8	98.9	–[a]
	50	104	87	81	91	12	97.8	
	75	106	83	89	93	12	100.0	
	100	95	81	72	83	12	89.2	
Positive control A (Polyurethane film with 0.1%-zinc diethyldithiocarbamate)	0.5	42	53	49	48	6	51.6	0.52
	1	0	0	0	0	0	0.0	
	2	0	0	0	0	0	0.0	
	5	0	0	0	0	0	0.0	
	10	0	0	0	0	0	0.0	
Positive control B (Polyurethane film with 0.25%-zinc dibutyldithiocarbamate)	40	96	101	93	97	4	104.3	61
	50	92	97	88	92	5	98.9	
	60	53	51	46	50	4	53.8	
	80	1	0	0	0	1	0.0	
	100	0	0	0	0	0	0.0	

a : No decreases were observed on the rate of relative colony formation, as a result that IC_{50} value cannot be estimated.

Supplementary Table 1

Colony-forming assay for cytotoxic evaluation

ROTA➔ Polyrotaxane-based gel
CNT➔ Multiwall carbon nanotube
IL➔ Ionic liquid (N,N-Diethyl-N-methyl-N-（2-methoxyethyl）ammonium tetrafluoroborate)
HEMA➔ Poly-hydroxyethyl methacrylate gel

A

Appendix 1 One-week implantation

Cell type/response	ROTA				ROTA-IL				ROTA-CNT				ROTA-IL-CNT			
	00101	00102	00103	00104	00101	00102	00103	00104	00101	00102	00103	00104	00101	00102	00103	00104
Polymorphonuclear cells	2	2	3	3	2	1	3	3	4	2	3	3	3	2	3	2
Lymphocytes	1	1	1	1	1	1	1	1	0	1	1	1	1	1	1	1
Plasma cells	0	0	0	0	0	0	0	0	0	0	0	0	0	0	0	0
Macrophages	3	3	2	2	2	2	2	2	2	3	3	3	2	3	3	3
Giant cells	0	0	0	0	0	1	0	0	1	1	1	0	1	1	0	1
Necrosis	1	2	2	2	1	2	2	3	2	1	2	2	1	1	2	1
SUB TOTAL (X2)	14	16	16	16	12	14	16	18	18	16	20	18	16	16	18	16
Neovascularisation	1	1	0	0	0	0	0	0	0	0	1	0	1	0	0	0
Fibrosis	2	2	1	1	1	1	1	1	1	1	1	1	1	1	1	1
Fatty infiltrate	0	0	0	0	0	0	0	0	0	0	0	0	0	0	0	0
SUB TOTAL	3	3	1	1	1	1	1	1	1	1	2	1	2	1	1	1
TOTAL	70				64				77				71			
AVERAGE	17.5				16.0				19.3				17.8			

Cell type/response	HEMA				HEMA-CNT				HEMA-IL-CNT				Au				Negative control			
	00101	00102	00103	00104	00101	00102	00103	00104	00101	00102	00103	00104	00101	00102	00103	00104	00101	00102	00103	00104
Polymorphonuclear cells	3	4	3	1	2	2	4	3	3	4	3	4	3	3	4	3	2	2	1	1
Lymphocytes	1	1	1	0	1	1	1	1	1	1	1	1	0	1	0	0	0	1	1	1
Plasma cells	0	0	0	0	0	0	0	0	0	0	0	0	0	0	0	0	0	0	0	0
Macrophages	2	3	2	2	3	3	3	2	3	3	3	3	2	3	3	2	3	3	2	2
Giant cells	0	0	0	0	0	0	0	0	0	0	0	0	0	0	0	0	0	0	0	0
Necrosis	1	2	2	1	2	2	2	1	2	2	2	1	1	2	2	2	2	2	1	2
SUB TOTAL (X2)	14	20	16	8	16	16	20	14	18	20	18	18	12	18	18	14	14	16	10	12
Neovascularisation	0	0	0	0	1	0	0	0	0	0	0	0	0	0	0	0	0	0	0	0
Fibrosis	1	1	1	2	1	1	1	1	1	1	1	1	1	2	1	1	0	1	0	1
Fatty infiltrate	0	0	0	0	0	0	0	0	0	0	0	0	0	0	0	0	0	0	0	0
SUB TOTAL	1	1	1	2	2	1	1	1	1	1	1	1	1	2	1	1	0	1	0	1
TOTAL	63				71				78				67				54			
AVERAGE	15.8				17.8				19.5				16.8				13.5			

B

Appendix 2 4-week implantation

Cell type/response	ROTA				ROTA-IL				ROTA-CNT				ROTA-IL-CNT			
	00105	00106	00107	00108	00105	00106	00107	00108	00105	00106	00107	00108	00105	00106	00107	00108
Polymorphonuclear cells	1	2	1	2	1	0	1	1	0	2	0	2	1	1	0	0
Lymphocytes	1	2	3	2	2	1	2	3	2	3	1	2	3	1	2	1
Plasma cells	0	1	1	0	0	0	1	0	0	0	0	0	0	1	0	0
Macrophages	3	3	4	2	3	3	3	3	3	3	3	3	3	2	3	3
Giant cells	1	0	0	0	0	1	0	0	2	1	1	1	1	0	1	1
Necrosis	0	0	1	0	1	1	0	1	0	1	1	0	1	1	0	0
SUB TOTAL (X2)	12	16	20	12	14	12	14	16	14	20	12	16	18	12	12	10
Neovascularisation	1	2	1	1	0	0	1	0	1	2	0	1	2	1	0	0
Fibrosis	2	2	2	1	1	1	2	1	2	2	1	2	2	1	1	2
Fattyinfiltrate	0	0	0	0	0	0	0	0	0	0	0	0	0	0	0	0
SUB TOTAL	3	4	3	2	1	1	3	1	3	4	1	3	4	2	1	2
TOTAL	72				62				73				61			
AVERAGE	18.0				15.5				18.3				15.3			

Cell type/response	HEMA				HEMA-CNT				HEMA-IL-CNT				Au				Negative control			
	00105	00106	00107	00108	00105	00106	00107	00108	00105	00106	00107	00108	00105	00106	00107	00108	00105	00106	00107	00108
Polymorphonuclear cells	2	1	0	1	2	2	1	0	1	3	1	2	2	1	2	2	0	0	0	0
Lymphocytes	2	2	2	1	2	3	1	2	3	3	3	3	3	3	3	2	0	0	0	0
Plasma cells	1	1	0	0	1	0	0	0	0	1	0	0	3	2	1	1	0	0	0	0
Macrophages	3	3	3	1	3	3	2	3	3	3	3	3	2	2	3	3	1	1	2	1
Giant cells	1	0	1	0	0	0	0	0	1	1	1	0	0	0	0	0	0	0	0	0
Necrosis	1	0	0	0	1	0	0	0	1	2	0	1	1	0	1	0	0	0	0	0
SUB TOTAL (X2)	20	14	12	6	18	16	8	10	18	26	16	18	22	16	20	16	2	2	4	2
Neovascularisation	1	0	0	0	2	2	0	0	2	2	2	2	2	1	0	0	0	0	0	0
Fibrosis	1	1	1	2	1	3	1	1	2	2	3	3	3	3	2	3	1	1	2	2
Fattyinfiltrate	0	0	0	0	0	0	0	0	0	0	0	0	0	0	0	0	0	0	0	0
SUB TOTAL	2	1	1	2	3	5	1	1	4	4	5	5	5	4	2	3	1	1	2	2
TOTAL	58				62				96				88				16			
AVERAGE	14.5				15.5				24.0				22.0				4.0			

Supplementary Table 2

More quantitative data analysed from the pathology grafts and a summary of the results 1

ROTA➔ Polyrotaxane-based gel
CNT➔ Multiwall carbon nanotube
IL➔ Ionic liquid (N,N-Diethyl-N-methyl-N-(2-methoxyethyl) ammonium tetrafluoroborate)
HEMA➔ Poly-hydroxyethyl methacrylate gel

	Average irritant ranking score	⊿ between test sample and control [a]	Non-irritant (0.0 up to 2.9)<br>Slight irritant (3.0 up to 8.9)<br>Moderate irritant (9.0 up to 15.0)<br>Severe irritant (>15)
1-week implantation			
Negative control	13.5	–	–
ROTA	17.5	4.0	Slight irritant
ROTA-IL	16.0	2.5	Non-irritant
ROTA-CNT	19.3	5.8	Slight irritant
ROTA-IL-CNT	17.8	4.3	Slight irritant
HEMA	15.8	2.3	Non-irritant
HEMA-CNT	17.8	4.3	Slight irritant
HEMA-IL-CNT	19.5	6.0	Slight irritant
Au	16.8	3.3	Slight irritant
4-week implantation			
Negative control	4.0	–	–
ROTA	18.0	14.0	Moderate irritant
ROTA-IL	15.5	11.5	Moderate irritant
ROTA-CNT	18.3	14.3	Moderate irritant
ROTA-IL-CNT	15.3	11.3	Moderate irritant
HEMA	14.5	10.5	Moderate irritant
HEMA-CNT	15.5	11.5	Moderate irritant
HEMA-IL-CNT	24.0	20.0	Severe irritant
Au	22.0	18.0	Severe irritant
a: Negative difference is recorded as zero.			

Supplementary Table 3

more quantitative data analysed from the pathology grafts and a summary of the results 2
